# Pre-service teachers' perceptions of physical, socioemotional and cognitive traits in gifted students: unveiling bias?

**DOI:** 10.3389/fspor.2024.1472880

**Published:** 2025-01-07

**Authors:** Carmen Ferrándiz, Mercedes Ferrando-Prieto, Álvaro Infantes-Paniagua, Maria Carmen Fernández Vidal, Rosa María Pons

**Affiliations:** ^1^ Department of Development and Educational Psychology, University of Murcia, Murcia, Spain; ^2^ Department of Physical Education, Arts Education and Music, University of Castilla-La Mancha, Albacete, Spain

**Keywords:** stereotypes, implicit theories, high ability students, pre-service teachers, physical attributes

## Abstract

**Introduction:**

Attitudes and beliefs guide our decision-making. In the educational context, prior research has noted the existence of prejudices and stereotypes among teachers that make it difficult to identify and care for gifted students. Stereotypes towards gifted students can hinder the identification and development of potential and the development of personality. This study examines Spanish pre-service teachers' stereotypical conceptions of gifted and non-gifted students focusing on physical appearance and athletic ability.

**Methods:**

Following a mixed methods research with between subjects design and using a convenience sampling, 455 last-year pre-service teachers enrolled during 2023–2024 in one of three university degrees or one Master's degree related to teacher training at University of Murcia were randomly assigned a vignette in order to rate the intellectual ability, motivation, prosociality, and physical characteristics of a fictitious 12-year-old student whose ability level (gifted/non-gifted) and gender (girl/boy), varied. Additionally, participants were asked to describe how they imagined the fictitious student's physical appearance. After exploring measurement invariance (SPSS AMOS 29), a MANCOVA was performed to compare the results across vignettes (SPSS 28). The study delves specifically into the physical characteristics attributed to gifted students through qualitative analysis addressing co-occurrence coefficients (Atlas.ti 9).

**Results:**

The results suggest that pre-service teachers described gifted students as more intelligent and with better physical attributes, especially gifted females. Furthermore, gifted students of both genders were defined as more intelligent, creative, and tall. They considered gifted girls to be attractive and gifted boys to be good at sports, highly fit, formally dressed, and wearing glasses.

**Discussion:**

The results are relevant as they allow a greater understanding of the perception of these students. As pointed out by pioneering studies in the field, implicit theories relate intelligence to physical appearance. This evidence could improve the training of future teachers, and therefore, the identification and assessment of gifted students from different areas.

## Introduction

The identification of gifted and talented students plays a key role in establishing educational policies and practices that respond to the needs of these students, monitor their progress, and enable the development of their full potential (1–3). Nevertheless, gifted identification remains an unsolved question. As Goodhew [(4), p. 8] states, “Identifying potentially gifted and talented students has never been an exact science”, and proof of that is the different models and criteria cited in the literature (5). What seems to be a consensus in the scientific community is the need for identification to be a comprehensive process in which different facets are taken into account and all interest groups—students, teachers and families—are included (6).

Relying solely on intelligence tests and IQ scores, though still prevalent, faces criticism in specialized literature. While IQ tests remain integral, testing all students is not feasible due to logistical constraints and ethical considerations. Hence, teachers' preliminary identification assumes significance, as they serve as gatekeepers, influencing access to tailored educational interventions (2). Teachers can nominate potentially gifted students, considering individual and contextual characteristics within the framework of talent (1).

However, this approach is not without drawbacks: when teachers nominate, several issues arise regarding the influence that perceptions, beliefs and stereotypes about the nature of giftedness and the characteristics of gifted students can have on the decision-making process (7–9), on expectations (10), on the way of interacting with these students (11), and on behaviour in general (12).

In this regard, despite the conceptual change regarding giftedness, switching from a unidimensional structure to a multidimensional structure of giftedness that include talents in different domains like sports (13) or arts (14), among others, a vision of giftedness associated with a high IQ, academic success in all curricular areas and difficulties in the socioemotional area still prevails in the general population, in part due to “it is easier to identify ‘the gifted’ by a score” [(15), p. 235]. This vision corresponds to the harmony/disharmony hypothesis (16). The harmony hypothesis says that gifted students have high academic potential and performance, as well as a socioemotional adjustment equal to or superior to that of their peers (17). In contrast, the second hypothesis maintains that high ability “comes as cost” [(18), p. 182] in the socioemotional sphere and in psychological adjustment (7, 19). But if, as Bergold et al. (20) points out, “decades of empirical research have falsified the disharmony hypothesis” (p. 1), one might wonder why the image of the gifted continues to be associated with such a hypothesis.

The answer should be sought in the implicit bias that people hold. “Implicit bias refers to prejudicial attitudes towards and stereotypical beliefs about a particular social group or members therein” [(21), p. 1,457], and these representations to determinate characteristics or traits influence prejudices and guide our behaviour (22). In the educational context, these biases impact how teachers perceive and respond to specific characteristics such as gender, physical attributes, socioeconomic status, emotional stability, academic performance, etc (8, 23).

Many of these stereotypes are present and perpetuated through mass media, so it is not surprising that many teachers are influenced by these preconceived ideas. Bergold et al. (20) demonstrated this hypothesis by assessing attitudes towards gifted individuals after exposing participants to two different versions of the same article about giftedness. In their study, participants were divided into two groups. Half read an article published in mass media about giftedness in which general assessments were made following a stereotypical view; additionally, some specific cases of gifted individuals were analysed in the article through interviews. The other half of the participants read a revised version of the same article. In this version, the characteristics of the interviewed gifted individuals remained the same, but general statements with stereotypes about gifted individuals “stating that gifted children as a rule develop normally and do not, on average, have more problems than other children” [(20), p. 79] were modified. Participants who read the revised article showed a more positive attitude towards gifted people.

These stereotypes are also present among professionals who work with gifted individuals (i.e., primary school teachers, secondary school teachers, and psychologists). In the study by Sánchez et al. (24), conducted in France, participants (general population and professionals) were asked to provide five characteristics that they associated with gifted children and/or adolescents. A lexical analysis to extract the core ideas given by the participants found that the general population associated giftedness with intellectual characteristics while teachers (in both primary and secondary education) were more concerned about their social adaptation, giving less importance to the academic area. As for psychologists, they focused on intrapersonal aspects related to high sensitivity and an intellectual predisposition (i.e., curiosity). This shows that biases persist despite being debunked by research [see (25–27)].

One approach used to assess stereotypes about gifted students consists of the use of vignettes (descriptions of scenarios involving a student) and the assessment of cognitive, motivational, and socioemotional characteristics, among others, of the student represented in said vignette (7, 10–12, 28). In this approach, each group of participants sees a vignette with subtle differences in the main character; so the effects of the character's skill level (gifted vs. non-gifted), gender (female vs. male) and age (8 vs. 9–15 years old) can be analysed. In different research using this approach, different character variables have been incorporated and others have been eliminated. In all cases, stereotypes referring to maladjustment and social skills are always included. Consistent results on maladaptation have been found: the gifted were always perceived as having worse adjustment than their peers (10–12, 28). Regarding social skills, European teachers (German and/or Belgian) perceived the gifted as less socially competent (10, 11, 28), while Australian teachers did not perceive differences between gifted and non-gifted (12, 28). Beyond ability, Australian teachers perceived that boys in both groups had worse social skills than girls in both groups and that girls in both groups were better adjusted than boys.

Baudson and Preckel (7) carried out a study with a sample of primary and secondary in-service and pre-service teachers, in which the description of a student represented in a vignette was requested, controlling three variables in the student to be described: skill level (gifted vs. average), age (8 vs. 15 years) and gender (girl vs. boy). The results obtained confirmed the stereotype of lack of harmony: teachers perceived those who were gifted as more open to new experiences, more introverted, less emotionally stable, and less agreeable, the gifted variable being the most influential in the assessment of personality traits. In the study by Baudson and Preckel (10), the motivation of the students was also analysed, finding that although adolescents tend to be evaluated as having low motivation, the 15-year-old gifted students were the ones who obtained higher motivation scores. The authors also found that years of teaching experience affected motivation ratings: new teachers tended to rate girls higher.

Matheis et al. (28) investigated enthusiasm for teaching various student profiles and teaching self-efficacy in German and Australian teachers. They found no differences in enthusiasm for teaching gifted vs. non-gifted students; however, in general, Germans preferred teaching girls and specifically showed greater enthusiasm for average ability girls compared to other profiles. Australian teachers also preferred teaching girls regardless of their ability level. Both groups of teachers had higher self-efficacy for teaching average ability students than gifted students and higher self-efficacy for teaching girls than boys.

In Weyns et al.'s (11) study, variables related to personality, anxious behaviour, expected teacher-child relationship, likability, and emotional demand were also analysed. It was found that gifted individuals were less extraverted, less agreeable, less emotionally stable, more open, more conscientious, and more anxious. Regarding the teacher-child relationship, no differences were found in closeness or dependency in their relationship with the hypothetical gifted child; however, teachers did expect more conflict. Gifted children were similar in likability to average ability children, but they were perceived as demanding more attention than the latter.

Additionally, Tan et al. (29) conducted a study to investigate the perceptions of 52 secondary education students regarding intelligence and differences in intelligence development at different ages, perceptions about giftedness and the development of giftedness, and how these perceptions relate to intelligence and giftedness. They used both a survey and a vignette task. Overall, participants perceived intelligence as mostly related to both 'school' and “non-school” intelligence, motivation, and knowledge and learning. In addition, students associated giftedness with intelligence, motivation, high ability, and academic achievement. Participants endorsed an incremental belief about giftedness and believed motivation mattered for developing giftedness. They also reported that giftedness could be developed by increasing motivation and learning, and that all students had potential. Students were slightly more certain that young children could increase intelligence, and that intelligence can grow through hard work. Compared with gifted participants, non-gifted participants were more likely to believe intelligence can grow across ages. Finally, gifted participants were more likely to associate intelligence with knowledge and learning and interests than non-gifted participants.

It is worth noting that the series of studies mentioned here focus on explicit stereotypes. “That is, they assessed intentionally edited rather than automatic responses” [(30), p. 1,164]. Such explicit measures are prone to the influence of social desirability and response biases. In contrast, the work by Preckel et al. (30) specifically focuses on studying implicit stereotypes. To do this, the authors used a series of rapid tasks where participants responded intuitively and with little time margin. First, participants were asked to familiarize themselves with photos of six students, three of whom were gifted. After this, a computer task was used in which the participants were exposed to one of the photos and then asked to assign a valence (positive or negative) to a specific adjective. There was a total of 28 adjectives, carefully selected, representing terms commonly used to describe gifted students, high-achieving students, and social maladjustment. Participants underwent trials with each of the photos and each of the adjectives in random order (a total of 168 trials). The results confirmed the disharmony hypothesis for gifted males but not for gifted females.

As can be seen, there is extensive research on stereotypes related to the gifted; however, physical stereotypes of gifted students have not been studied as much (17). An exception is the work of Carman (23), in which prospective teachers were asked to imagine a gifted person. When mentioning their physical characteristics, 81 per cent of the participants described a non-athletic student; 40 per cent described them as short, and 60 per cent as tall.

Despite the stereotype of conceiving intelligent individuals as awkward, clumsy, weak, or physically unattractive [(31), cited in (32)], psychologists have intuited since the early studies in measuring intelligence that mental traits are somehow linked to physical traits. “Good mental development accompanies good physical growth during childhood” [(33), p. 40].

Baldwin [(33), p. 7] considers that “the term “gifted” should always be qualified by such words as mentally, aesthetically, and physically”, and proposes chronological age observation as a method of identification. Referring to his earlier research, he found that high-intelligence students were taller than lower-intelligence students. Thus, in early research, a good number of articles attempted to prove this intuition [see (34, 35)].

Some works in this period considered whether the differences in height, weight and body size were due to the family of origin: gifted students usually came from educated and socially well-positioned families. To answer this question, Laycock and Caylor (36) took a group of 81 gifted children and their respective siblings in order to compare the gifted with the non-gifted. Their results indicated that there were no differences between gifted and non-gifted in physical measurements (weight, height, bi-acromial and bi-iliac diameters, and leg circumference). However, there was a bias as some participants in the non-gifted siblings group showed higher IQ scores than those in the gifted group.

Also working with pairs of siblings, Chamrad et al. (37) found that mothers tend to describe their gifted children (if they are the eldest) as more physically attractive; when athletic skills were compared, the authors found that the non-gifted developed these more as compensation.

In 2014, a study in South Korea selected gifted male students in mathematics and science, as well as non-gifted students. It was found that the gifted students were significantly taller than the non-gifted students and had lower body mass index and percentage of body fat. However, the authors (38) did not find differences in physical fitness between gifted and non-gifted students. It is worth mentioning that gifted students attending the Korea Science Academy (KSA) were required to complete a sports programme, which could include taekwondo, trekking, or marathon.

The study by Hormazabal-Peralta et al. (39) examined the physical conditions and the level of weekly physical activity of a sample of 71 gifted adolescents in Chile. Although the study does not compare these students' results with those of the normal population, it offers interesting data regarding weekly physical activity. Most gifted students (69.86%) engaged in more than two hours of scheduled physical activity, while only 30.14 per cent engaged in less than two hours. Students who engaged in more than two hours of physical activity showed lower fat mass index (FMI) and body fat percentage (BF%), and higher muscle mass percentage (MM%). The study also presented some gender differences in terms of certain anthropometric measures: weight, height, BMI, BF%, MM%, and FMI; as expected, boys were on average taller than girls. It was also found that girls had higher obesity rates than boys.

The research by Infantes-Paniagua et al. (40) showed no statistically significant differences in the levels of weekly physical activity reported by gifted and non-gifted students in secondary education grades. A similar result was obtained by Otero Rodríguez et al. (41), who found no differences between gifted and non-gifted individuals in physical fitness (general fitness, cardiorespiratory fitness, muscular strength, speed and agility, flexibility) or in weekly physical activity; however, they did confirm that gifted girls tended to be more sedentary than non-gifted girls. In the study by Çakiroğlu (42), physical traits and the motor ability of gifted and non-gifted children aged 9 to 13 without a history of professional sports were compared. No differences were found in weight, height, or body mass. However, differences were found in psychomotor ability measures such as vertical jump, standing broad jump, right-left grip power, right-left visual and auditory reaction time, 30 seconds of sit-ups, and stability, favouring the gifted individuals.

Some studies have also suggested that physical attractiveness is related to high abilities. Kanazawa and Kovar (43) proposed a model to explain the link between intelligence and the physical attributes of beauty. According to these authors, the most competent men have higher social status, which allows them to mate with more attractive women (p. 239). Since both intelligence and beauty are inherited, this means that both go hand in hand throughout generations. But is it true that gifted people are more attractive? Hollingworth's (44) study compared the physical attractiveness of gifted individuals using photographic portraits of gifted and non-gifted adolescent boys and girls and asking some teachers who did not know them to estimate the physical attractiveness of the people portrayed. In this study, the faces of the highly intelligent were shown to be more attractive (more beautiful) than the faces of members of the ordinary group, all other things being equal. Years later, Zebrowitz et al. (45) conducted a similar study: different participants were asked to rate the attractiveness of different photographed people of different ages for whom different data, including IQ, had been collected. They found that, at all stages of life, except for elderly people, perceived attractiveness correlated around .2 with the IQ of the person photographed.

It is worth highlighting the research by Luftig and Nichols (46), in which classmates were asked to rate the attributes of gifted students. The authors found that gifted boys were rated more attractive than their peers, while gifted girls did not differ in physical attractiveness from non-gifted girls. This is one of the few studies (to the best of our knowledge) that distinguishes between boys and girls.

Additionally, it is interesting to mention Jackson et al. (47) meta-analysis, which verified that physically attractive adults and children were perceived as more intellectually competent than their less attractive peers. By the year 2000, Langlois et al.'s (48) meta-analysis sought correlates of different measures with physical beauty, finding that more attractive children displayed greater intelligence/performance competence, whereas attractive adults showed only slightly more intelligence.

Moreover, although anorexia nervosa has been associated with giftedness in girls due to their high degree of perfectionism, the work of Godor et al. (49) reveals that gifted individuals are less susceptible to emotional eating; furthermore, they experience less social anxiety than the normative sample.

In 1986, Benbow (50) conducted an extensive study with 416 families of gifted students who responded to a series of questions regarding the physical characteristics and health of these students. She found that among extremely mathematically and/or verbally precocious students (top 1 in 10,000 in reasoning ability), the following three physiological characteristics were found at high frequencies: left- or mixed-handedness, asthma and other allergies, and myopia (50). A subsequent study by Lubinski and Humphreys (51) examined a large sample of students across the United States who had participated in Project Talent, a project aimed at identifying gifted individuals. The participating students were extensively evaluated in different areas; among them, 23 items related to their overall health. The authors divided the sample into three groups: mathematical talents, privileged students (with a high socioeconomic status), and ordinary students. They found that mathematical talents had better health indicators, including allergies, although they were more likely to wear glasses.

Using the UK biobank (a biomedical database containing genetic, lifestyle and health information, as well as biological samples from half a million UK participants), Williams et al. (52) found that people with high IQ (two standard deviations above the mean) showed greater frequency of suffering from allergies, eczema and myopia.

Considering the previous research, it can be summarized that gifted students are not necessarily less well-adjusted than non-gifted, and regarding their physical characteristics, they are similar to non-gifted: it might even be stated that they tend to be perceived more attractive and more healthy than non-gifted students. But do teachers perceive gifted students as the research pictures them?

The aim of this study was to examine Spanish pre-service teachers' stereotypical conceptions of gifted and non-gifted students in terms of cognition, socioemotional, and physical attributes, with a special focus on physical appearance and athletic ability, from a two-fold quantitative and qualitative approach. This work will benefit the field of giftedness and talent studies as it will shed light on the presence of stereotypes among pre-service teachers, thereby contributing to a better understanding that can be incorporated into their training. Additionally, the study will expand prior knowledge by providing a qualitative perspective on the conceptions of pre-service teachers. All of this will lead to improved procedures for the identification and support of high intellectual abilities.

## Materials and methods

### Study design

This is a descriptive study using a mixed-methods approach. Sampling was carried out by convenience and a between-subject design was used.

### Participants

The participants were 455 last-year pre-service teachers (66.2% female, 33.2% male, 0.7% other) between 18 and 51 years old (*M* = 23.30, *SD* = 4.34) from three Bachelor's degrees (17.4% Bachelor's degree in Early Years Education, 30.5% Bachelor's degree in Primary Education, 10.1% Bachelor's degree in Physical Activity and Sports Sciences) and one Master's degree (42% Master's degree in Secondary Education and Baccalaureate, Vocational Training, Language Teaching, and Artistic Education) from two campuses at University of Murcia in the southeast of Spain (Table 1).

**Table 1:** Participants’ demographic data.

Group	Categories		%
Gender	Male		33.2%
	Female		66.2%
	Other		0.7%
Mean age (years)			23.30
Educational degree	Bachelor's degree in Early Years Education		17.4%
	Bachelor's degree in Primary Education		30.5%
	Bachelor's degree in Physical Activity and Sports Sciences		10.1%
	Master's degree in Secondary Education and Baccalaureate, Vocational Training, Language Teaching, and Artistic Education		42%
Specialization	Bachelor's degree in Early Years Education	Early Years	84.8%
		Dual degree (Early Years and Primary Education)	15.2%
	Bachelor's degree in Primary Education	Special Education	19.4%
		Music	10.8%
		English	15.1%
		French	12.2%
		Physical Education	35.3%
		Dual degree (Early Years and Primary Education)	4.3%
		No mention	7.2%
	Bachelor's degree in Physical Activity and Sports Sciences	Physical Education	100%
	Master’ degree	Biology and Geology	9.4%
		Technology	3.7%
		Music	1%
		Mathematics	5.2%
		Spanish Language and Literature	9.9%
		Foreign Language (English)	13.6%
		Geography and History	5.8%
		Physics and Chemistry	7.9%
		Foreign Language (French)	8.9%
		Biosanitary studies	9.9%
		Management	7.3%
		Philosophy	4.7%
		Classical Languages (Latin and Greek)	5.8%
		Physical Education	3.7%
		No mention	3.1%

The research used a convenience sampling. The participants were randomly assigned a vignette to rate the intellectual ability, motivation, prosocial behaviour and physical characteristics of a fictitious 12-year-old student whose ability level (gifted/non-gifted) and gender (girl/boy) varied. Additionally, participants were asked to describe how they imagined their corresponding fictitious student's physical appearance.

### Instruments

#### Sociodemographic characteristics

Participants were asked their age, gender and teaching specialization (e.g., Special Education, Music, Foreign Languages, Physical Education) through an online questionnaire.

#### Vignettes and questionnaire

**Figure 1:**
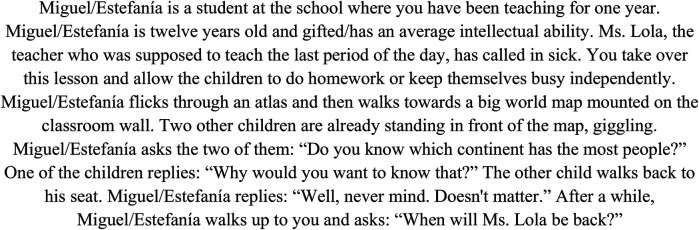
Vignette model adapted from Matheis et al. (12, 28). The participants were shown a Spanish adaptation of the vignette.

The vignettes were adapted from previous studies (12, 28) and were used as a stimulus for participants to rate the characteristics of the fictional students depicted in the vignettes. Each vignette comprised a brief description of a fictional student in an everyday school situation (Figure 1). After participants had read the vignette, they were administered a questionnaire consisting of 15 items on a six-point Likert-type scale (1 = *completely false*, 6 = *completely true*). The first 12 items are an adaptation to Spanish of the questionnaire used in Baudson and Preckel (10). This contains questions related to the dimensions of intellectual ability (three items; fluid reasoning, given that high cognitive potential is a crucial characteristic in both scientific and popular conceptions of giftedness); motivation (three items; involvement in class); prosocial behaviour (three items; positive behaviour in social interactions); and maladjustment (three items; general rule-breaking behaviour, not limited to social interactions). Three further items based on the Spanish short version of the Self-Description Questionnaire-II (53, 54) were included in order to address physical attributes (appearance/attractiveness and athletic ability). Also, inspired by Carman's (23) work, an open-ended qualitative item was added in which participants were asked to describe how they imagine the student (stimulus) described in the vignette in terms of physical appearance, fitness and athletic ability [“How do you imagine Estefanía/Miguel physically (his/her physical appearance, as well as his/her fitness and athletic ability)? Please, describe him/her in 15 words or fewer”].

Once participants had answered the questionnaire on their corresponding vignette, they were asked to rate their prior experience with gifted students and their knowledge on giftedness using a five-point Likert scale (1 = *no experience/knowledge*, 5 = *extensive experience/knowledge*).

### Procedure

The present study followed an between-subject design in which each participant was randomly assigned one of four vignette types (ability level x gender). This procedure resulted in four groups of approximately equal size (the number of participants for the four vignette conditions was *n_gifted boy_* = 112, *n_non−gifted boy_* = 114, *n_gifted girl_* =114, and *n_non−gifted girl_* = 115). Each participant rated only one fictitious student on intellectual ability, motivation, prosocial behaviour, maladjustment and physical attributes. The instruments were completed through the Murcia university's survey application. The research was conducted with the prior consent of the participants, who were randomly coded to make their responses anonymous. Participation was voluntary and took between 94 and 995 seconds (*M* = 405.13, *SD* = 118.06). The procedures were assessed and approved by the Ethics Committee on Research University of Murcia (reference: M10/2023/056).

### Statistical analysis

**Figure 2:**
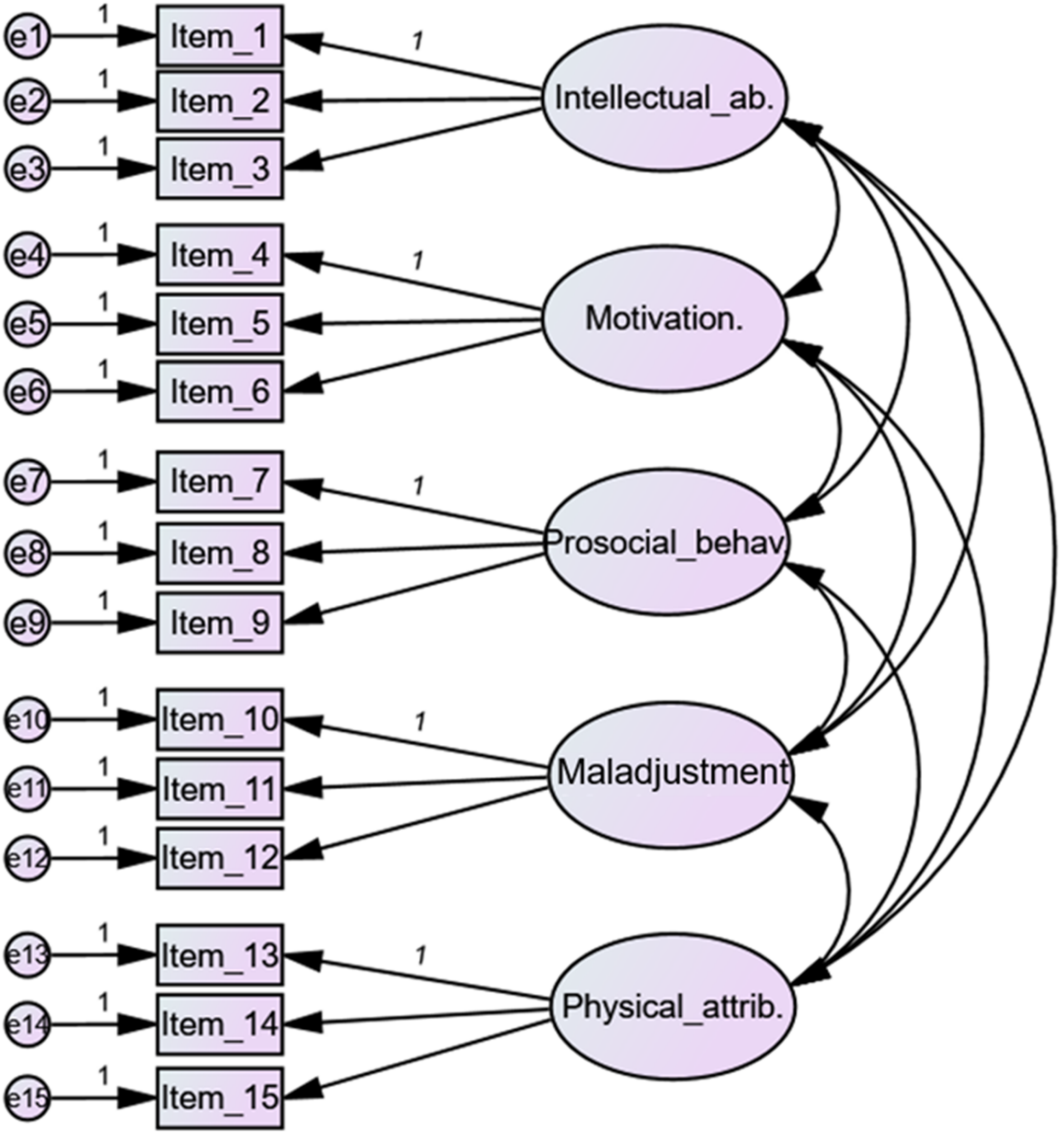
Path analysis structure for MI testing.

Both quantitative and qualitative analysis were conducted. On the one hand, following previous works on this matter [i.e., (12, 28)], preliminary analyses were conducted to test the measurement invariance (MI) of the questionnaire's quantitative items through multi-group confirmatory factor analysis (MGCFA). Before this analysis, statistical assumptions of good fit were confirmed for each group in the configural model. More concretely, chi-squared by degrees of freedom ratio [*χ*^2^/*df*; <3 as acceptable (55)]; and root mean square error of approximation (RMSEA; <.05 or <.06 as good and <.08 as acceptable) were explored as absolute fit indexes; Tucker-Lewis index (TLI) and comparative fit index (CFI; both >0.90 acceptable, >0.95 optimal) were employed as incremental or comparative fit indexes; finally, the Akaike's information criterion (AIC) was explored as a parsimony-adjusted index (56). Since the configural MI model (Figure 2) fit could be considered good in general terms (see Supplementary Tables S1–S3), configural, metric, scalar, and strict MI models were compared following the methods stated by Byrne (57) and Crowson (58). The reference values for the MGCFA were non-significant *Δχ*^2^ (58), *Δ*CFI ≤ .01 (59), and *Δ*RMSEA < .015 (60). Analyses were conducted using SPSS AMOS v.29.

To compare the results across vignettes, a multivariate analysis of covariance (MANCOVA) was performed, including participants' gender, prior experience and previous knowledge about giftedness as covariates. Five dependent variables were used: intellectual ability, motivation, prosocial behaviour, maladjustment, and physical attributes. The independent variable was the vignette condition. Preliminary assumption testing was conducted to check for normality, linearity, univariate and multivariate outliers, homogeneity of variance covariance matrices, and multicollinearity, with no serious violations noted, except for the normality assumption; however, considering the large sample (*N* > 30), it could be assumed according to the central limit theorem (61). By assessing the Mahalanobis distance, two outliers were identified and removed for this statistical analysis. The effect size assessment (proportion of the variance in the dependent variable that can be explained by the independent variable) of each of the results was obtained by using the partial eta squared (*η*^2^) proposed by Cohen (62), where .01–.06 is a small effect; .06–.14 is a moderate effect and a value more than .14 is a large effect. Bonferroni correction (*p* < .01) was applied to the MANCOVA. The analyses were carried out using IBM SPSS Statistics version 28.0.1.1.

Qualitative analyses were carried out using Atlas.ti 9. For item 16 [“How do you imagine Estefanía/Miguel physically (her/his physical appearance, as well as her/his fitness and athletic ability)? Please, describe her/him within 15 word or less”], two researchers each coded a randomly assigned 50 per cent of each vignette's qualitative responses. They followed an independent open coding procedure, consisting of creating codes for each characteristic or idea expressed by the participants. The first stage yielded 490 codes. These codes were then revised by merging them when different codes referred to the same attribute (e.g., “beautiful” and “pretty”) (63). This procedure, carried out simultaneously by both researchers, reduced the number of codes to 185. These codes were organized into 16 main categories and 33 subcategories, any discrepancy was discussed for alignment with the categories. Additionally, 44 codes were introduced to identify each participant's vignette case and other relevant information (e.g., gender, degree, self-reported experience and knowledge of giftedness). Frequencies of codes, categories and subcategories were explored (Supplementary Tables S4–S7). To focus on the main differences between the four vignettes, the co-occurrence coefficient (*c*) was used only for those whose frequencies summed at least 1 per cent of the times. The *c*-coefficient indicates the strength of the relationship between two codes within a range from 0 (no relationship) to 1 (the strongest relationship). Its calculation is based on approaches borrowed from quantitative content analysis (64).

## Results

### Quantitative results

#### Measurement invariance testing across vignette conditions

According to the MI testing (Table 2), despite the significant *Δχ^2^* (*p* = .014), partial scalar MI was found for the whole questionnaire model (*Δ*CFI = 0.008; *Δ*RMSEA = 0.001). Only when intercepts of the items 2 and 7 were set free, the parameter tested showed a better fit than the full scalar invariance model (Supplementary Table S2). Therefore, comparison across the four vignette types was feasible.

**Table 2:** Tests for MI for the questionnaire across the four vignette groups.

Model	*χ^2^*	*df(χ^2^)*	*p*(*χ^2^*)	CFI	RMSEA	Comparison	*Δχ^2^*	*Δdf(χ^2^)*	*Δp*(*χ^2^*)	*Δ*CFI	*Δ*RMSEA
1. Configural	427.972	320	<.001	0.951	0.027						
2. Metric	462.992	350	<.001	0.949	0.027	2 vs. 1	35.020	30	.242	0.002	0.000
3. Full scalar	535.419	380	<.001	0.93	0.030	3 vs. 2	72.427	30	.000*	0.019	0.003
4. Partial scalar	504.544	374	<.001	0.941	0.028	4 vs. 2	41.552	24	.014*	0.008	0.001
5. Strict	603.237	395	<.001	0.906	0.034	5 vs. 4	98.693	21	.000*	0.035	0.006

* *p* < .05; *Δ*RMSEA < .015; *Δ*CFI < .01.

#### Differences among preservice teachers in relation to vignette conditions

Table 3 presents the means and standard deviations for the dependent variables (intellectual ability, motivation, prosocial behaviour, maladjustment, and physical attributes) across the four vignette conditions. Descriptive statistics are shown for prior experience and previous knowledge of giftedness. Scale reliability was estimated by computing Cronbach's alpha and McDonald's omega, also listed in Table 3.

**Table 3:** Descriptive and reliability statistics.

Variable	*α*	*ω*	Gifted *n* = 226 *M* (*SD*)	Average *n* = 227 *M* (*SD*)	Boy *n* = 226 *M* (*SD*)	Girl *n* = 227 *M* (*SD*)	Gifted girl *n* = 114 *M* (*SD*)	Gifted boy *n* = 112 *M* (*SD*)	Non-gifted girl *n* = 113 *M* (*SD*)	Non-gifted boy *n* = 114 *M* (*SD*)
Intellectual ability^a^	0.815	0.818	4.787 (0.817)	4.387 (0.870)	4.587 (0.850)	4.587 (0.885)	4.9181 (0.764)	4.6548 (0.852)	4.2537 (0.877)	4.520 (0.847)
Motivation^a^	0.685	0.707	4.178 (1.048)	4.055 (1.034)	4.045 (1.069)	4.188 (1.012)	4.3421 (0.968)	4.012 (1.104)	4.032 (1.036)	4.078 (1.037)
Prosocial behaviour^a^	0.776	0.785	3.874 (0.918)	3.966 (0.879)	3.849 (0.896)	3.991 (0.898)	3.994 (0.884)	3.753 (0.940)	3.988 (0.917)	3.944 (0.843)
Maladjustment^a^	0.545	0.614	2.146 (0.793)	2.342 (0.846)	2.299 (0.849)	2.218 (0.798)	2.032 (0.747)	2.619 (0.824)	2.3481 (0.819)	2.336 (0.875)
Physical attributes^a^	0.827	0.848	3.287 (1.002)	3.014 (0.891)	3.119 (0.927)	3.182 (0.987)	3.383 (1.033)	3.190 (0.966)	2.9794 (0.900)	3.050 (0.886)
Prior experience with giftedness	–	–	1.86 (1.005)	1.88 (0.988)	1.87 (0.987)	1.86 (1.006)	1.81 (1.021)	1.91 (0.991)	1.92 (0.992)	1.83 (0.986)
Previous knowledge of giftedness	–	–	2.50 (0.881)	2.44 (0.912)	2.50 (0.915)	2.45 (0.878)	2.49 (0.914)	2.52 (0.849)	2.41 (0.841)	2.48 (0.980)

Skewness: −0.759, −0.151; kurtosis: -.398, 1.253 (*N* = 453).

^a^ Number of items = 3.

A one-way 2 × 2 between-groups multivariate analysis of covariance (MANCOVA) was performed on five dependent variables: intellectual ability, motivation, prosocial behaviour, maladjustment, and physical attributes. Independent variables were vignette condition ability (gifted and non-gifted) and gender (girl and boy). In addition, the gender of participants, prior experience and previous knowledge were entered as covariates. Before the test, we checked the assumptions (outliers with Mahalanobis distance, linearity, multicollinearity, univariate and multivariate normality, homogeneity, and homoscedasticity). Box test was not significant (*F Box* = .909; *p* = .646). Thus, conditioned matrices homogeneity of variance/covariance was met. Also, Levene's test was used to examine the equality of variances between the groups. The results confirmed the equality of variances for intellectual ability (*f* = .290; *p* = .832), motivation (*f* = .215; *p* = .886), prosocial behaviour (*f* = .329; *p* = .805); maladjustment (*f* = 1.140; *p* = .332), and physical attributes (*f* = .808; *p* = .490). The results of the MANCOVA using Wilk's lambda as a criterion are presented in Table 4.

**Table 4:** MANCOVA of vignette conditions’ effects on intellectual ability, motivation, prosocial behaviour, maladjustment, and physical attributes, controlling for participants’ gender, prior experience with giftedness and previous knowledge of giftedness.

	Multivariate				Univariate														
					Intellectual ability			Motivation			Prosocial behaviour			Maladjustment			Physical attributes		
	Wilks's *λ*	*F* ^e^	*p*	*η^2^_p_*	*F* ^f^	*p*	*η^2^_p_*	*F* ^f^	*p*	*η^2^_p_*	*F* ^f^	*p*	*η^2^_p_*	*F* ^f^	*p*	*η^2^_p_*	*F* ^f^	*p*	*η^2^_p_*
Intercept ^a^	0.223	308.774	<.001	0.777	659.770	0.000	0.597	326.369	0.000	0.423	292.034	0.000	0.396	208.935	0.000	0.319	156.964	0.000	0.260
Corrected model^b^	–	–	–	–	6.238	0.000	0.077	1.401	0.212	0.019	3.137	0.005	0.040	2.674	0.015	0.035	4,979	0.000	0.063
Intercept^b^	–	–	–	–	659.770	0.000	0.597	326.369	0.000	0.423	292.034	0.000	0.396	208.935	0.000	0.319	156.964	0.000	0.260
Gender participant^c^	0.966	3.136	0.009*	0.034	0.130	0.718	0.000	0.058	0.810	0.000	4.659	0.031	0.010	0.739	0.390	0.002	10.733	0.001*	0.024
Prior experience^c^	0.983	1.154	0.184	0.017	0.153	0.696	0.000	0.766	0.382	0.002	0.002	0.962	0.000	0.362	0.548	0.001	6,148	0.014	0.014
Previous knowledge^c^	0.968	2.889	0.014	0.032	0.182	0.670	0.000	0.007	0.934	0.000	7.691	0.006*	0.017	4.324	0.038	0.010	0.408	0.523	0.001
Vignette^d^	0.869	4.245	<.001*	0.046	12.411	<.001*	0.077	2.531	0.057	0.017	1.839	0.139	0.012	3.548	0.015	0.023	4.503	0.004*	0.029

* significance values based on Bonferroni correction (*p* < .01).

^a^ multivariate.

^b^ between-subjects’ effect.

^c^ covariate.

^d^ fixed factor (vignettes: gifted girl, non-gifted girl, gifted boy, non-gifted boy).

^e^ *df*: intercept *F*(5, 442); covariates: *F*(5, 442); fixed factor (vignette): *F*(15, 1,220.569).

^f^ *df*: corrected model *F*(6, 452); intercept *F*(1, 446); covariates: *F*(1, 446); fixed factor (vignette): *F*(3, 446).

There was a statistically significant difference between the vignette conditions' groups on the combined dependent variables after controlling for gender, prior experience and previous knowledge of giftedness: *F*(15, 1,220.569) = 4.245, *p* < .001, Wilks' *Λ* = .869, partial *η^2^* = .046. Also the covariates gender of participants—*F*(5, 442) = 3.136, *p* = .009, Wilks' *Λ* = .966, partial *η^2^* = .043—and previous knowledge of giftedness—*F*(5, 442) = 2.889, *p* = .014, Wilks' *Λ* = .968, partial *η^2^* = .032—were, respectively, significative and marginally significant.

**Figure 3:**
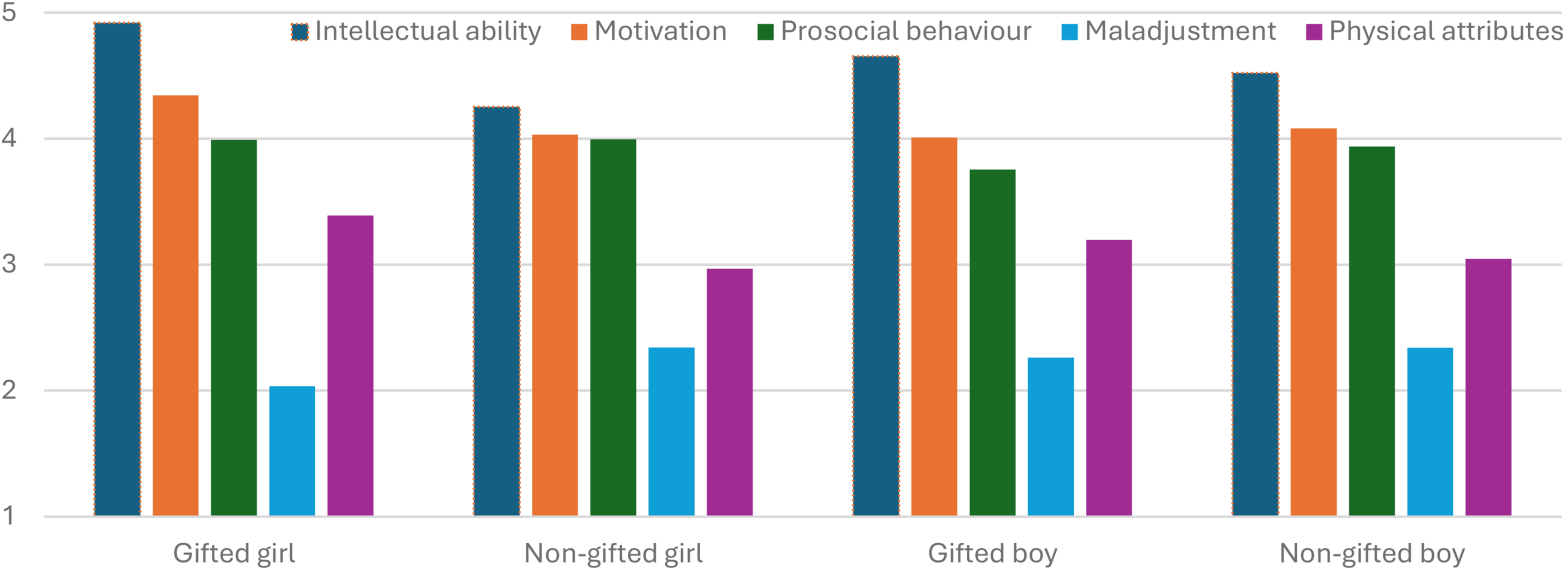
Profile plot of estimated marginal means on dependent variables by vignette groups. *c*ovariates appearing in the model are evaluated at the following values: gender (girl: 2, boy: 1, other: 0) = 1.66; prior experience with giftedness = 1.87; previous knowledge of giftedness = 2.47.

Analyses of covariance (ANCOVA) were conducted for each dependent variable as follow-up tests for MANCOVA. The follow-up ANCOVA test indicated that the covariate of gender of participants affected dependent variable physical attributes: *F*(1, 446) = 10.733, *p* = .001, partial *η^2^* = .024. The previous knowledge covariate affected the dependent variable prosocial behaviour: *F*(1, 446) = 7.691, *p* = .006, partial *η^2^* = .017. The fixed factor (vignettes) had a significant effect on intellectual ability: *F*(3, 446) = 12.411, *p* < .001, partial *η^2^* = .077; and on physical attributes: *F*(3, 446) = 4.503, *p* < .004, partial *η^2^* = .029, after controlling for gender of participants, prior experience and previous knowledge. The effect of vignette groups on maladjustment was marginally significant: *F*(3, 446) = 3.548, *p* = .015, partial *η^2^* = .023 (see Table 4). Figure 3 displays the profile plot of estimated marginal means of intellectual ability, motivation, prosocial behaviour, maladjustment, and physical attribute for the four groups of vignette conditions (gifted girl, non-gifted girl, gifted boy, and non-gifted boy). *Post hoc* Bonferroni tests were subsequently conducted to inspect specific between-group differences. There were statistical differences for intellectual ability between gifted girls and non-gifted girls, as well as between gifted girls and non-gifted boys, favouring gifted girls in both cases. Also, differences were found between gifted boys' and non-gifted girls' vignettes, favouring gifted boys. Pre-service teachers rated gifted students as significantly intellectually more able than average ability students. For physical attributes, the results showed differences favouring gifted girls vs. non-gifted girls (see Table 5).

**Table 5:** Post-hoc comparison Bonferroni test.

			95% Confidence interval		
Variable	Group differences	Cohen's *d*	Lower	Upper	*p*
Intellectual ability	Gifted girl > non-gifted girl	0.808	0.536	1.078	*<*.001
	Gifted girl > non-gifted boy	0.493	0.229	0.756	.002
	Gifted boy > non-gifted girl	0.464	0.198	0.728	.002
Physical attributes	Gifted girl > non-gifted girl	0.417	0.153	0.679	.009

### Qualitative results

Considering the main aim of this study, we will focus especially on physical attributes. Since there were numerous codes regarding physical attributes (86 codes), only those that were mentioned at least 1 per cent of the time are included in Table 6. Other non-physical attributes were also addressed by the participants (98 codes); similarly, those that were mentioned at least 1 per cent of the time can be found in Table 7. The complete lists of codes referring to physical and non-physical attributes can be found in the Supplementary Tables S4–S7.

**Table 6:** Categories, subcategories and codes with frequency and percentage of mentioned physical attributes.

Main category	Subcategory	Code	All		Gifted girl (*n* = 113)			Non-gifted girl (*n* = 113)			Gifted boy (*n* = 112)			Non-gifted boy (*n* = 114)			Gifted (*n* = 225)			Non-gifted (*n* = 227)		
			*k*	%	*k*	%	*c*	*k*	%	*c*	*k*	%	*c*	*k*	%	*c*	*k*	%	*c*	*k*	%	*c*
Appearance	Athleticism		5	0.29	0	0	N/A	1	20	N/A	4	80	N/A	0	0	N/A	4	80	N/A	1	20	N/A
	Attractiveness		28	1.61	9	32.14	N/A	5	17.86	N/A	6	21.43	N/A	8	28.57	N/A	15	53.57	N/A	13	46.43	N/A
	Care		9	0.52	4	44.44	N/A	2	22.22	N/A	2	22.22	N/A	1	11.11	N/A	6	66.67	N/A	3	33.33	N/A
	Clothes		36	2.06	6	16.67	N/A	11	30.56	N/A	11	30.56	N/A	8	22.22	N/A	17	47.22	N/A	19	52.78	N/A
	Eyes		34	1.95	9	26.47	N/A	13	38.24	N/A	6	17.65	N/A	6	17.65	N/A	15	44.12	N/A	19	55.88	N/A
	Face		179	10.30	44	24.58	N/A	47	26.26	N/A	46	25.7	N/A	42	23.46	N/A	90	50.28	N/A	89	49.72	N/A
		Wearing glasses	153	8.77	31	20.26	.13	41	26.8	.18	44	28.76	.20	37	24.18	.16	75	49.02	.25	78	50.98	.26
	Hair		221	12.70	63	28.51	N/A	65	29.41	N/A	52	23.53	N/A	41	18.55	N/A	115	52.04	N/A	106	47.96	N/A
		Blonde hair	29	1.66	10	34.48	.08	10	34.48	.08	7	24.14	.05	2	6.897	.01	17	58.62	.07	12	41.38	.05
		Dark hair	108	6.19	28	25.93	.14	22	20.37	.11	29	26.85	.15	29	26.85	.15	57	52.78	.21	51	47.22	.18
	Height		229	13.1	61	26.64	N/A	50	21.83	N/A	55	24.02	N/A	63	27.51	N/A	116	50.66	N/A	113	49.34	N/A
		Short	79	4.53	16	20.25	.09	26	32.91	.16	14	17.72	.08	23	29.11	.14	30	37.97	.11	49	62.03	.19
		Standard	64	3.67	14	21.88	.09	13	20.31	.08	15	23.44	.09	22	34.38	.14	29	45.31	.11	35	54.69	.14
		Tall	86	4.93	31	36.05	.18	11	12.79	.06	26	30.23	.15	18	20.93	.10	57	66.28	.22	29	33.72	.10
	Others		48	2.75	13	27.08	N/A	13	27.08	N/A	13	27.08	N/A	9	18.75	N/A	26	54.17	N/A	22	45.83	N/A
		Standard global appearance	38	2.18	11	28.95	.08	10	26.32	.07	12	31.58	.09	5	13.16	.03	23	60.53	.10	15	39.47	.06
	Physical development		6	0.34	1	16.67	N/A	3	50	N/A	1	16.67	N/A	1	16.67	N/A	2	33.33	N/A	4	66.67	N/A
	Size		185	10.60	47	25.41	N/A	37	20	N/A	45	24.32	N/A	56	30.27	N/A	92	49.73	N/A	93	50.27	N/A
		Slim	118	6.77	28	23.73	.14	19	16.1	.09	31	26.27	.16	40	33.9	.21	59	50.00	.21	59	50.00	.21
		Standard body size	42	2.41	11	26.19	.08	12	28.57	.08	10	23.81	.07	9	21.43	.06	21	50	.09	21	50	.08
	Skin		18	1.03	6	33.33	N/A	5	27.78	N/A	4	22.22	N/A	3	16.67	N/A	10	55.56	N/A	8	44.44	N/A
Athletic competence	Athletic competence		45	2.58	10	22.22	NA	7	15.56	NA	11	24.44	NA	17	37.78	NA	21	46.67	NA	24	53.33	NA
Behaviour^a^	Athletic		11	0.63	3	27.27	NA	0	0	NA	3	27.27	NA	5	45.45	NA	6	54.55	NA	5	45.45	NA
Fitness	Fitness		56	3.21	10	17.86	NA	7	12.5	NA	20	35.71	NA	19	33.93	NA	30	53.57	NA	26	46.43	NA
		Low physical fitness	18	1.03	2	11.11	.02	3	16.67	.02	4	22.22	.03	9	50	.07	6	33.33	.03	12	66.67	.05
		Standard physical fitness	21	1.20	6	28.57	.05	3	14.29	.02	5	23.81	.04	7	33.33	.05	11	52.38	.05	10	47.62	.04
Global trait	Global trait	Standard person	26	1.49	3	11.54	.02	4	15.38	.03	7	26.92	.05	12	46.15	.09	10	38.46	.04	16	61.54	.07
Health^a^	Health		7	0.40	0	0	NA	0	0	NA	5	71.43	NA	2	28.57	NA	5	71.43	NA	2	28.57	NA
Interests^a^	Sports		13	0.75	4	30.77	NA	1	7.692	NA	3	23.08	NA	5	38.46	NA	7	53.85	NA	6	46.15	NA
Skills/abilities^a^	Physical		2	0.11	0	0	NA	2	100	NA	0	0	NA	0	0	NA	0	0	NA	2	100	NA
Unknown/cannot determine		Unknown/cannot determine	30	1.72	6	20.00	.04	8	26.67	.06	12	40.00	.09	4	13.33	.03	18	60.00	.08	12	40.00	.05

*k*: frequency of codifications. *c*: co-occurrence coefficient. N/A: not applicable.

^a^ These categories are not itemized into codes because these codes showed a frequency <1%.

**Table 7:** Categories, subcategories and codes with frequency and percentage of other mentioned attributes.

Main category	Subcategory	Code	All		Gifted girl (*n* = 113)			Non-gifted girl (*n* = 113)			Gifted boy (*n* = 112)			Non-gifted boy (*n* = 114)			Gifted (*n* = 225)			Non-gifted (*n* = 227)		
			*k*	%	*k*	%	*c*	*k*	%	*c*	*k*	%	*c*	*k*	%	*c*	*k*	%	*c*	*k*	%	*c*
Behaviour	Academic		24	1.38	6	25.00	N/A	7	29.17	N/A	6	25.00	N/A	5	20.83	N/A	12	50.00	N/A	12	50.00	N/A
	Social		13	0.75	2	15.38	N/A	2	15.38	N/A	6	46.15	N/A	3	23.08	N/A	8	61.54	N/A	5	38.46	N/A
Feelings/mental state	Feelings/mental state		6	0.34	1	16.67	N/A	0	0.00	N/A	4	66.67	N/A	1	16.67	N/A	5	83.33	N/A	1	16.67	N/A
Interests	Academic/intellectual	Academic/intellectually engaged	28	1.61	6	21.43	.04	9	32.14	.07	8	28.57	.06	5	17.86	.04	14	50.00	.06	14	50.00	.06
	Global		33	1.89	3	9.09	N/A	9	27.27	N/A	9	27.27	N/A	12	36.36	N/A	12	36.36	N/A	21	63.64	N/A
		Likes learning	25	1.43	3	12.00	.02	7	28.00	.05	8	32.00	.06	7	28.00	.05	11	44.00	.05	14	56.00	.06
	Others		8	0.46	3	37.50	N/A	2	25.00	N/A	2	25.00	N/A	1	12.50	N/A	5	62.50	N/A	3	37.50	N/A
Personality	CON High		23	1.32	6	26.09	N/A	4	17.39	N/A	6	26.09	N/A	7	30.43	N/A	12	52.17	N/A	11	47.83	N/A
		CON High (code)	16	0.92	5	31.25	.04	3	18.75	.02	4	25.00	.03	4	25.00	.03	9	56.25	.04	7	43.75	.03
	CON Low		2	0.11	0	0.00	N/A	0	0.00	N/A	1	50.00	N/A	1	50.00	N/A	1	50.00	N/A	1	50.00	N/A
		CON Low (code)	2	0.11	0	0.00	.00	0	0.00	0	1	50.00	.01	1	50.00	.01	1	50.00	.00	1	50.00	.00
	EXT High		8	0.46	3	37.50	N/A	3	37.50	N/A	2	25.00	N/A	0	0.00	N/A	5	62.50	N/A	3	37.50	N/A
		EXT High (code)	8	0.46	3	37.50	.03	3	37.50	.03	2	25.00	.02	0	0.00	.00	5	62.50	.02	3	37.50	.01
	EXT Low		68	3.90	13	19.12	N/A	20	29.41	N/A	15	22.06	N/A	20	29.41	N/A	28	41.18	N/A	40	58.82	N/A
		EXT Low (code)	67	3.84	12	17.91	.07	20	29.85	.13	15	22.39	.09	20	29.85	.12	27	40.30	.10	40	59.70	.16
		Shy/introverted	56	3.21	8	14.29	.05	16	28.57	.10	12	21.43	.08	20	35.71	.13	20	35.71	.08	36	64.29	.15
	KIND High		59	3.38	19	32.20	N/A	16	27.12	N/A	11	18.64	N/A	13	22.03	N/A	30	50.85	N/A	29	49.15	N/A
		KIND High (code)	46	2.64	14	30.43	.10	12	26.09	.08	8	17.39	.05	12	26.09	.08	22	47.83	.09	24	52.17	.10
		Kind	27	1.55	8	29.63	.06	8	29.63	.06	4	14.81	.03	7	25.93	.05	12	44.44	.05	15	55.56	.06
	KIND Low		2	0.11	0	0.00	N/A	0	0.00	N/A	2	100.00	N/A	0	0.00	N/A	2	100.00	N/A	0	0.00	N/A
		KIND Low (code)	1	0.06	0	0.00	.00	0	0.00	.00	1	100.00	.01	0	0.00	.00	1	100.00	.00	0	0.00	.00
	NEU High		32	1.83	4	12.50	N/A	11	34.38	N/A	10	31.25	N/A	7	21.88	N/A	14	43.75	N/A	18	56.25	N/A
		NEU High (code)	29	1.66	4	13.79	.03	11	37.93	.08	8	27.59	.06	6	20.69	.04	12	41.38	.05	17	58.62	.07
	NEU Low		3	0.17	1	33.33	N/A	0	0.00	N/A	1	33.33	N/A	1	33.33	N/A	2	66.67	N/A	1	33.33	N/A
		NEU Low (code)	3	0.17	1	33.33	.01	0	0.00	.00	1	33.33	.01	1	33.33	.01	2	66.67	.01	1	33.33	.00
	OE High		53	3.04	15	28.30	N/A	13	24.53	N/A	10	18.87	N/A	15	28.30	N/A	25	47.17	N/A	28	52.83	N/A
		OE High (code)	49	2.81	13	26.53	.09	13	26.53	.09	9	18.37	.06	14	28.57	.09	22	44.90	.09	27	55.10	.11
		Curious	38	2.18	9	23.68	.06	11	28.95	.08	6	15.79	.04	12	31.58	.09	15	39.47	.06	23	60.53	.10
	OE Low		1	0.06	0	0.00	N/A	1	100.00	N/A	0	0.00	N/A	0	0.00	N/A	0	0.00	N/A	1	100.00	N/A
		OE Low (code)	1	0.06	0	0.00	.00	1	100.0	.01	0	0.00	.00	0	0.00	.00	0	0.00	.00	1	100.00	.00
Self-perception	Self-perception		6	0.34	1	16.67	N/A	1	16.67	N/A	0	0.00	N/A	4	66.67	N/A	1	16.67	N/A	5	83.33	NA
Skills/abilities	Academic		18	1.03	5	27.78	N/A	1	5.56	N/A	4	22.22	N/A	8	44.44	N/A	9	50.00	N/A	9	50.00	NA
	Coping		4	0.23	0	0.00	N/A	1	25.00	N/A	2	50.00	N/A	1	25.00	N/A	2	50.00	N/A	2	50.00	NA
	Intellectual/cognitive		58	3.33	24	41.38	N/A	10	17.24	N/A	13	22.41	N/A	11	18.97	N/A	37	63.79	N/A	21	36.21	NA
		Intelligent	44	2.52	18	40.91	.13	8	18.18	.05	9	20.45	.06	9	20.45	.06	27	61.36	.11	17	38.64	.07
	Socioemotional		35	2.01	7	20.00	N/A	12	34.29	N/A	8	22.86	N/A	8	22.86	N/A	15	42.86	N/A	20	57.14	NA
		Low social skills	31	1.78	7	22.58	.05	10	32.26	.07	7	22.58	.05	7	22.58	.05	14	45.16	.06	17	54.84	.07
Social	Social		35	2.01	6	17.14	N/A	9	25.71	N/A	7	20.00	N/A	13	37.14	N/A	13	37.14	N/A	22	62.86	NA

*k*: frequency of codifications. *c*: co-occurrence coefficient.

N/A, not applicable; CON, conscientiousness; EXT, extraversion; KIND, kindness; NEU, neuroticism; OE, openness to experience.

Firstly, the answers of the 452 participants resulted in 185 codes that were mentioned 1,744 times. It is important to note that 30 participants (1.72%) mentioned that they were unable to describe or determine any physical or non-physical attribute of the character presented in their corresponding vignette. Regarding the appearance, 13.1 per cent of the time the participants referred to the height, showing similar percentages across the vignettes and codes. The gifted students were seen as tall more frequently than the non-gifted students (*c* = .22 v. *c* = .10). Hair (12.70%) and size (10.6%) were the following most mentioned attributes. It can be highlighted that the non-gifted boy was regarded as slim more frequently (*c* = .21) than the other vignettes, although the gifted cases also showed a non-significant frequency within this category (girl: *c* = .14, boy: *c* = .16). Additionally, attributes regarding face were mentioned, with a notable number of references to “wearing glasses” (*k* = 153) in the four vignettes; this condition was most mentioned for the gifted boy (*c* = .20) in comparison with the others (gifted girl: *c* = .13; non-gifted girl: *c* = .18; non-gifted boy: *c* = .16). Despite this, it must be noted that two of the four participants that explicitly mentioned “not wearing glasses” referred to the gifted boy (see Supplementary Table S3). Furthermore, although the “attractiveness” subcategory showed low frequency (*k* = 28), participants more frequently referred to the gifted girl with terms coded into this subcategory than in the other cases. More concretely, 45.45 per cent of the time the gifted girl was referred to as “attractive”, while the non-gifted girl was not explicitly referred to as such but was considered “non-attractive” in 41.67 percent of cases (Supplementary Table S2), being the vignette with most mentions for this code (*k* = 5). Among the codes regarding clothing, the reference to “formal clothes” (*k* = 14) must be highlighted, which was especially present in the gifted boy vignette (*c* = .05). Finally, there were 38 mentions coded as “standard global appearance” with similar frequency across the vignettes, except for the non-gifted boy, where the frequency was lower (*c* = .03).

The second main category within physical attributes was athletic competence. In this case, the most common ideas were coded as “not good at sports” (*k* = 14) and “good at sports” (*k* = 12). Both vignettes showing girls received similar frequency in those codes (*k* = 4 and *k* = 2, respectively), while for boys' vignettes, the non-gifted boy was more frequently assessed as “not good at sports” (*k* = 6) and the gifted boy “good at athletics” (*k* = 4). Also, it must be highlighted that both non-gifted vignettes (80.00%) were regarded as “clumsy” more frequently than the gifted ones (especially the boy: 50.00%). The third main category focused on fitness (*k* = 56). In this case, participants described the vignettes' fictitious students as showing “standard” (*k* = 21), “low” (*k* = 18) or “high” (*k* = 17) physical fitness. The first was referred to equally across the groups, the non-gifted girl receiving the lowest number of mentions (*k* = 3). The non-gifted boy was most often referred to as showing low fitness (*k* = 9, *c* = .07), while the gifted boy was most often referred to as showing high fitness (*k* = 11, *c* = .09). Finally, when participants referred to vignettes as “normal” or “standard” without mentioning any specific attribute (e.g., appearance, fitness, athletic competence, etc.), these comments were coded as “global trait-standard person” (*k* = 26; 1.49%). This code showed a different frequency for each group (gifted girl: *k* = 3; non-gifted girl: *k* = 4; gifted boy: *k* = 7; non-gifted boy: *k* = 12).

Alongside physical attributes, participants referred to a series of non-physical attributes that were also coded and analysed. Among the seven categories established for organizing these codes, the largest referred to personality traits that participants attributed to the vignettes' fictional characters (*k* = 251). These traits were categorized according to the Big Five model of personality by following the list of adjectives included in the works of John and Srivastava (65). The personality trait most frequently referred to was low extraversion, with terms like “shyness” or “timidity” (*k* = 67), which were employed for the non-gifted characters (59.70%, *c* = .16) more frequently than for the gifted ones (40.30%, *c* = .10). Also, traits coded as “high openness to experience” were frequent, but in a similar way across the groups (*k* = 13–14, *c* = .09), except for fewer mentions for the gifted boy (*k* = 9, *c* = .06). Participants also referred to the high kindness of the fictional characters with similar frequency (*k* = 12–14, *c* = .08–.10), except for the gifted boy (*k* = 8, *c* = .05). Furthermore, looking at the total traits coded into “neuroticism”, most of the descriptions (*k* = 29) pointed to a high level, especially for the non-gifted girl (*k* = 11, *c* = .08) and noticeable for the gifted boy (*k* = 8, *c* = .06). There were fewer mentions of this trait for the gifted girl (*k* = 4, *c* = .03). Finally, a few participants mentioned a trait coded as “conscientiousness” (*k* = 18), most of them referring to a high level with similar frequency across groups (*k* = 3–5, *c* = .02–.04).

Abilities other than physical were also mentioned by the participants. Most were related to the intellectual or cognitive domains (*k* = 58), such as being creative, highly able in different areas or having a high level of attention. The most common attribute within this subcategory was intelligence (*k* = 44), which was notably more referred to within the gifted cases (61.36%) in comparison with the non-gifted students (38.64%). This was highlighted specially for the gifted girl (*k* = 18, *c* = .13) compared to the others (*k* = 8–9, *c* = .05–.06). Regarding socioemotional abilities, participants frequently referred to low skills equally across groups (*k* = 7, *c* = .05), except for more references in the case of the non-gifted girl (*k* = 10, *c* = .07). Also, participants commented on the interests that the fictional characters might have. A huge majority mentioned interests related to intellectual or academic domains (*k* = 28) despite the group; notwithstanding, the non-gifted girl (*k* = 9, *c* = .07) and gifted boy (*k* = 8, *c* = .06) slightly received more mentions than the others (gifted girl: *k* = 6, *c* = .04; non-gifted boy: *k* = 5, *c* = .4). Similarly, participants frequently mentioned that these fictional students like learning (*k* = 7–8, *c* = .05–.06), apart from the gifted girl (*k* = 3, *c* = .02).

Finally, mentions of characters' behaviours were also coded and, although most referred to the academic subcategories (*k* = 24), these covered a variety of themes (e.g., asking questions, participating in class, getting bored in class, etc.), with no clear differences. The only point to be highlighted is that the non-gifted girl was considered “teacher-dependent” (*k* = 5, *c* = .04) more frequently than the others (*k* = 1–2, *c* = .01–.02). Additionally, ideas that considered the social life of these fictional characters from the point of view of relationships were grouped into the “social” category (*k* = 35). None of the individual codes within this category showed a frequency higher than 1 per cent of the total; notwithstanding, it should be pointed out that both girls (70.00%, *c* = .03) were referred to as socially rejected slightly more frequently than boys (30.00%, *c* = .01). Also, comments regarding the scarcity of friends (*k* = 6) and not being popular (*k* = 7) were more common than the reverse notions such as having many friends (*k* = 1 for non-gifted female). It is of interest that the participants only mentioned bullying (*k* = 2) or referred to characters in terms of “freak” or “nerd” in few cases (*k* = 7), but all of them in the case of male students.

## Discussion

The study we have presented delves into the stereotypes and preconceived ideas that future teachers may hold about gifted students. Although some studies, such as that by Berezovskaya et al. (66), found that the majority (75%) of definitions of giftedness provided by teachers included positive aspects and only 3 per cent included negative aspects, research continues to highlight the existence of stereotypes regarding giftedness (7, 10, 12, 20, 28).

While previous studies have focused mainly on socioemotional aspects, in this work we have also specifically investigated some physical attributes that may be associated with high ability. Although this dimension is hardly studied, we think that its analysis is interesting since we are “embodied” and our body is our means of interaction with the environment and our first “letter of introduction” to others. Our physique represents our most evident features. For example, “Obese people are judged to be lazy and incompetent” [(67), p. 1,970]. A person with poorly groomed teeth will tend to denote both personal neglect as well as low socioeconomic status (since they cannot afford to go to the dentist). Even more, “disembodied” views of mind that separate cognition from the body are less accepted nowadays (68). It is inevitable that we form opinions about the characteristics of others based on the first thing we perceive: their physical appearance. These inferences occur spontaneously and quickly and influence the decisions we make (69). For this reason, we think that knowing what physical stereotypes are usually associated with high ability is relevant to understanding whether or not these can influence the identification of students.

Our results are consistent with classical research on the anthropomorphic characteristics and physical capabilities of the gifted [i.e., (33, 44)]. Furthermore, our participants tend to perceive gifted students as socially better adjusted, contradicting some previous research on prejudice about gifted students in which the vignette instrument has been used which has found results that support the disharmony hypothesis, finding that gifted students have worse adjustment than average students (7, 10, 12, 28). Our results are in line with those of Siegle and Powell (9) and Siegle et al. (70), who reported that teachers tend to equate giftedness with non-stereotypical (unexpected) perceptions.

As the work of Matheis et al. (28) shows, some differences in teachers' beliefs are found depending on the country they come from. In this sense, Oh et al. (71) found that occidental countries (particularly Latin countries) tend to perceive gifted students as more socially competent than Asian countries (Vietnam and South Korea).

Participants in our study were also asked to describe the student in the vignette. This approach allowed us better to understand the implicit beliefs held by future teachers. When answering a questionnaire, individuals express explicit beliefs and there may be a tendency to respond within socially acceptable norms. When pre-service teachers articulate their description of the child, they imagine such a situation, and we can extract some implicit beliefs that are not consciously acknowledged by the participants themselves (30).

We would like once again to highlight the importance of the use of vignettes and the type of questions selected. These questions and the situation presented have allowed us more accurately to explore the teachers' preconceived ideas, since previous research such as that by Pinnelli et al. (72) shows that when faced with a general open question (“what idea do you have about intellectual giftedness?”), teachers tend to respond according to the definitions extracted from the literature rather than offering their own vision of the construct.

**Figure 4:**
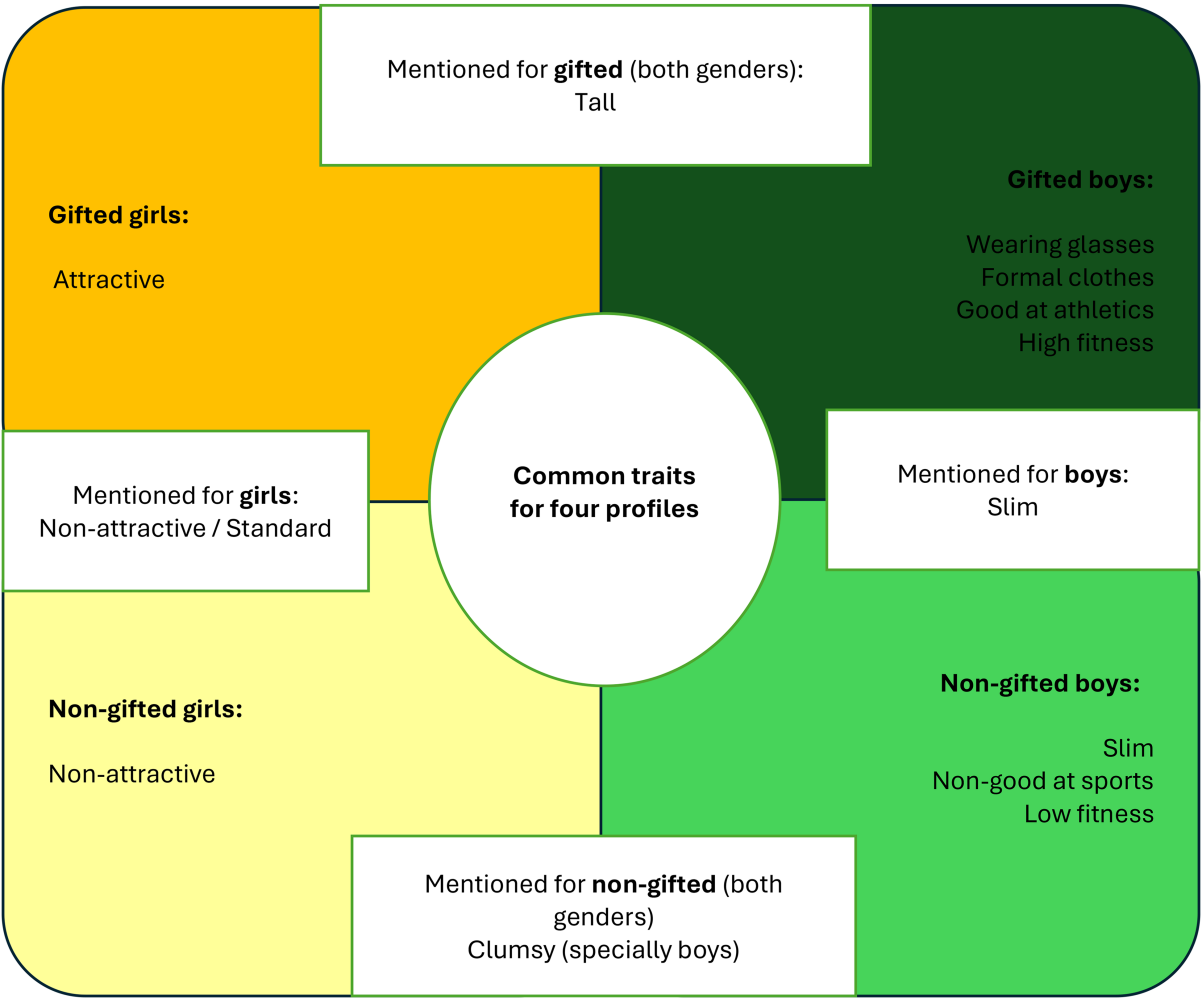
Summary of physical attributes in participants’ descriptions.

In our study, no differences were found between the two approaches (i.e., implicit/explicit). The ideas that were mentioned most when describing the gifted students represented in the vignettes were: tall (both genders); the boys were mentioned as being good at athletics, having high fitness, wearing formal clothing, and wearing glasses; the girls were mentioned as being attractive (see Figure 4).

Descriptions of non-gifted students included terms such as “clumsy” and “slim” (both genders); in the case of boys, references were made to “normal” or “ordinary”, not being good at sports and having low fitness.

Based on the adjectives that were used most frequently to describe each student's profile, we can extrapolate that, in general, participants perceive gifted students as having better physical attributes than non-gifted students. This finding contradicts the results obtained by Carman (23), in which gifted students were perceived as non-athletic. In Carman's study, some characteristics associated with the gifted can be seen, but as no comparison was made with non-gifted, we cannot see differential characteristics of these students.

**Figure 5:**
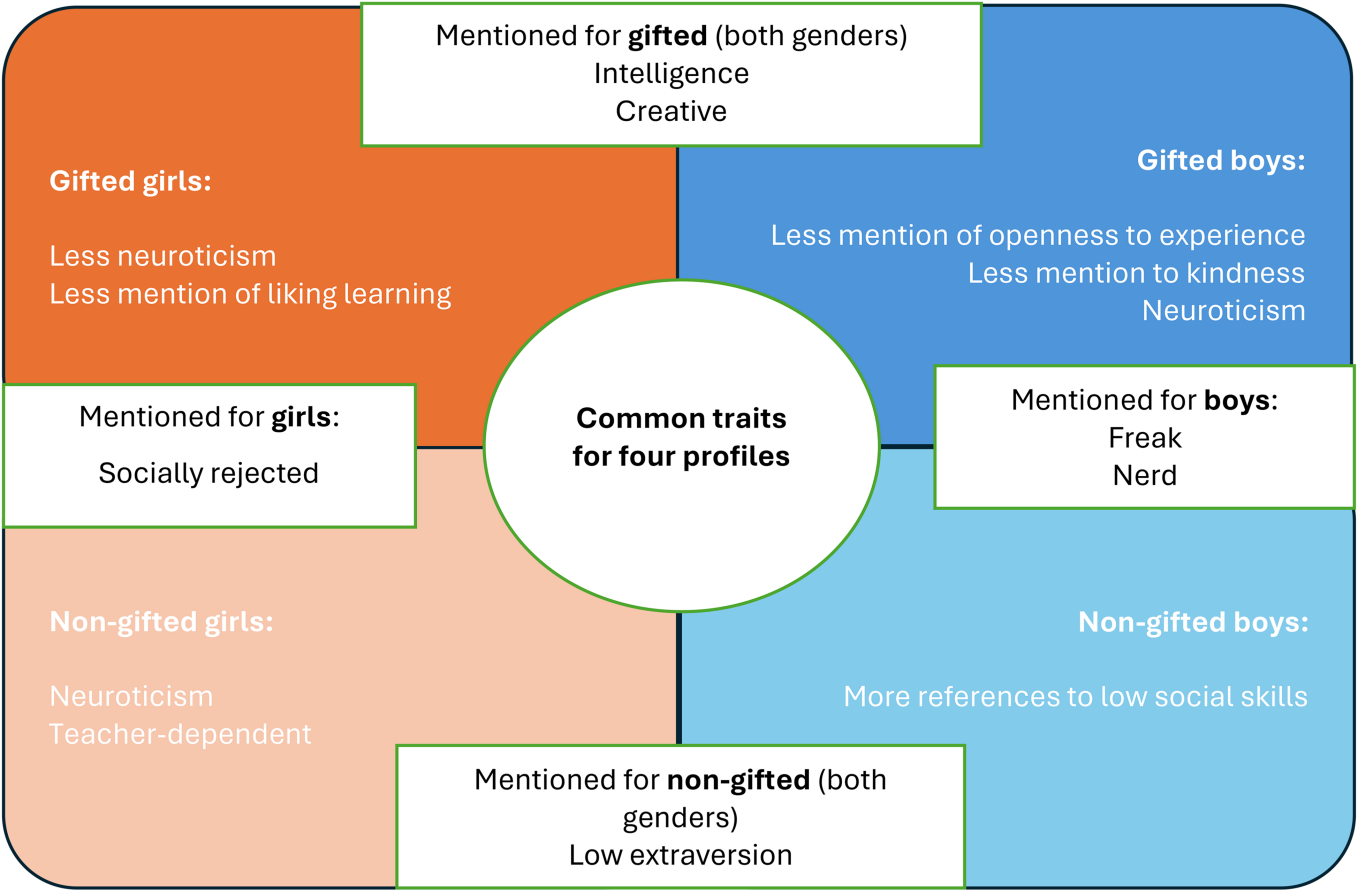
Summary of non-physical attributes in participants’ descriptions.

Regarding psychological aspects, the cognitive and creative domains were much mentioned for the gifted profiles; some differences in personality were also mentioned, neurotic traits being mentioned more times for non-gifted girls and gifted boys (see Figure 5).

Our results are different from those of Weyns et al. (11), whose hypothetical gifted child was described as less extraverted, less agreeable, less emotionally stable, more open, more conscientious, and more anxious. No mention was made of motivational aspects or curiosity, nor of academic achievement, contrary to Tan et al.'s (29) research. In our study, the less favoured profile is non-gifted girls, who tend to be perceived as neurotic, with low extraversion and socially rejected. In addition, they are described as physically non-attractive.

Our results could indicate the existence of a halo effect, whereby an overall positive or negative impression of a person influences the perception of specific traits. “The halo effect is often used in the positive perception of someone such as the perception that a child is gifted rules out all possible negative connotations that this label might bring. However, the halo effect also can have the opposite connotation” [(73), p. 203].

This phenomenon also occurs in previous studies, such as in the doctoral thesis by Roa Bañuelos (74), who asked teachers from different levels to describe a student with high abilities. The researcher found that “The most frequent responses are related to the possession of high levels of general intelligence, learning and work capacity, as well as creativity (teachers) or motivation (students); they are attributed boredom, frustration, and demotivation in class, and difficulty in interpersonal relationships with peers, being strange and lonely” [(74), p. 480].

It is worth mentioning that our participants were in many cases aware of the stereotypes that were put into play when responding to the questionnaire, despite the purpose of the research being concealed. Thus, some participants openly expressed that “the student's characteristics could not be assessed, since the information offered in the vignette was brief”, and some directly alluded to the fact that they were going to answer following the stereotypes:

Intellectual abilities do not influence a person's physical appearance. I consider them to be inverse aspects. There is often a perception of highly capable individuals as ‘nerdy’, with poor physical appearance and social skills, although highly developed cognitively. However, I believe there is no correlation (Participant 4027646, female).

If we follow stereotypes, she will be the typical girl with glasses, who no one approaches and they call her a nerd (Participant 4025622, female).

Almost all participants in this study may have pictured Miguel as an insecure and weak boy, both emotionally and physically (shy, slender, perhaps wearing glasses …) (Participant 4021526, male).

## Conclusions

Our results, based on a questionnaire format, confirm the harmony hypothesis. Differences were observed between groups in the intellectual dimension, particularly among gifted girls, which is expected given that the definition of giftedness itself includes or implies higher intelligence. Furthermore, gifted students of both genders were perceived as more intelligent, creative, and tall. High-ability students were also seen as having superior physical attributes, including greater physical competence and attractiveness. Pre-service teachers described gifted girls as attractive and gifted boys as athletic, highly fit, formally dressed, and often wearing glasses.

### Limitations of the study

This work has some limitations that need to be pointed out. Firstly, the sample has been selected through a non-random procedure. Also, the number of women is substantially higher than that of men. This is due to the types of degree analysed. Finally, although this work can provide knowledge for the design of teacher training programmes, the fact that the sample of participants is made up of students means that the influence of variables from the educational reality is not considered. These are students in their final year of training who have not yet been exposed to the reality of the classroom. In addition, the Spanish context regarding gifted education may be different from other contexts, as gifted education is mostly tackled as a transversal topic. In this regard, the work of Liesa Orús et al. (75) as well as Barrera-Algarín et al. (76) expose an analysis of training hours about giftedness in different teacher training degrees in Spain and an analysis of pre-service teachers' knowledge regarding giftedness.

### Implications of the study

In light of our results and comparing them with previous research in our country [e.g., Tourón et al. (77)], we can say that pre-service teachers are increasingly better informed about high abilities (giftedness and talent) and myths have been debunked. This may be due to increased research in this area and greater dissemination or transfer of scientific knowledge to the general population. It is worth mentioning that the educational approach in Spain is based on inclusive education: this approach is said to benefits not only students with disabilities or special needs but also their typically developing peers, as it promotes understanding, empathy, and respect for diversity.

The study delves especially into the physical characteristics attributed to gifted students, using a quantitative and qualitative approach. The results are relevant as they allow greater understanding of the idea we have about these students. It is revealing that, as pointed out by pioneering studies in the field, reviewed in the introduction, implicit theories relate intelligence to physical appearance.

### Future directions

Although stereotypes and teachers' perceptions of gifted students have been widely addressed in the literature, the diversity of results found, both in favour of the harmony hypothesis and against it, makes it necessary to carry out meta-analyses that shed light on this matter. Furthermore, clarification regarding the influence of variables linked to teachers (gender, previous experience, previous knowledge…) is relevant, as well as the types of perception analysed (explicit vs. implicit).

## Data Availability

The datasets presented in this study can be found in online repositories. The names of the repository/repositories and accession number(s) can be found below: Ferrándiz García C, Ferrando Prieto M, Infantes-Paniagua Á, Fernández Vidal MC, Pons Parra RM. Pre-service teachers' perceptions of gifted students [data set]. *Zenodo* (2024). https://doi.org/10.5281/zenodo.13121175.
