# Supplementary material for: Pre-service teachers' perceptions of physical, socioemotional and cognitive traits in gifted students: unveiling bias?
Source: Front Sports Act Living. 2025 Jan 7;6:1472880. doi: 10.3389/fspor.2024.1472880 (PMC11747620; doi:10.3389/fspor.2024.1472880)
Supplement: Supplementary file 1 [file Table1.docx]

Supplementary Material

**Table S1**

*Independent model fit for the four vignette groups*

| **Group** | ***N*** | ***χ^2^*** | ***df(χ^2^)*** | *χ^2^/* ***df*** | **RMSEA** | **TLI** | **CFI** | **AIC** |
| --- | --- | --- | --- | --- | --- | --- | --- | --- |
| Gifted girl | 114 | 80.459 | 80 | 1.006 | .007 | .999 | .999 | 160.459 |
| Nongifted girl | 115 | 122.422 | 80 | 1.530 | .068 | .904 | .927 | 202.422 |
| Gifted boy | 112 | 104.029 | 80 | 1.300 | .052 | .948 | .960 | 184.029 |
| Nongifted boy | 114 | 121.063 | 80 | 1.513 | .067 | .886 | .913 | 201.063 |
| Configural model (four vignettes) | 455 | 427.972 | 320 | 1.337 | .027 | .936 | .951 | 747.972 |

**Table S2**

*Comparison of models’ fit against testing intercepts against full scalar invariance model’s fit*

| **Parameter tested** | ***χ^2^*** | ***df(χ^2^)*** | **Δ*χ^2^*** | **Δ*df(χ^2^)*** | ***p*** |
| --- | --- | --- | --- | --- | --- |
| Intercept 1 | 524.402 | 377 | 11.017 | 3 | .012 |
| Intercept 2 | 522.132 | 377 | 13.287 | 3 | .004* |
| Intercept 3 | 532.779 | 377 | 2.64 | 3 | .450 |
| Intercept 4 | 530.887 | 377 | 4.532 | 3 | .209 |
| Intercept 5 | 531.260 | 377 | 4.159 | 3 | .245 |
| Intercept 6 | 526.337 | 377 | 9.082 | 3 | .028 |
| Intercept 7 | 517.833 | 377 | 17.586 | 3 | .001* |
| Intercept 8 | 534.349 | 377 | 1.07 | 3 | .784 |
| Intercept 9 | 530.839 | 377 | 4.58 | 3 | .205 |
| Intercept 10 | 534.353 | 377 | 1.066 | 3 | .785 |
| Intercept 11 | 528.049 | 377 | 7.37 | 3 | .061 |
| Intercept 12 | 534.950 | 377 | 0.469 | 3 | .926 |
| Intercept 13 | 530.485 | 377 | 4.934 | 3 | .177 |
| Intercept 14 | 526.512 | 377 | 8.907 | 3 | .031 |
| Intercept 15 | 524.912 | 377 | 10.507 | 3 | .015 |
| *Note:* * *p* < .01. It shows a better fit of the model freeing the parameter tested than the full scalar invariance model. | | | | | |

**Table S3**

*Correlation analysis between variables*

| **Variables** | **1** | **2** | **3** | **4** | **5** | **6** | **7** |
| --- | --- | --- | --- | --- | --- | --- | --- |
| 1. Intellectual ability | 1 |  |  |  |  |  |  |
| 2. Motivation | .283^**^ | 1 |  |  |  |  |  |
| 3. Prosocial behavior | .279^**^ | .095^*^ | 1 |  |  |  |  |
| 4. Maladjustment | -.275^**^ | -.230^**^ | -.178^**^ | 1 |  |  |  |
| 5. Physical attributes | .179^**^ | .119^*^ | .186^**^ | -.102^*^ | 1 |  |  |
| 6. Prior experience | .001 | .041 | .050 | -.005 | .109^*^ | 1 |  |
| 7. Previous knowledge | -.004 | .023 | .133^**^ | -.095^*^ | .017 | .378^**^ | 1 |
| *Note*. ∗*p* < .05. ∗∗*p* < .01 (2-tailed). | | | | | | | |

**Table S4**

*Categories, subcategories and codes with frequency and percentage of mentioned physical attributes by vignette*

|  |  |  | **All** | | **Gifted girl**  **(*n* = 113)** | | | **Non-gifted girl**  **(*n* = 113)** | | | **Gifted boy**  **(*n* = 112)** | | | **Non-gifted boy**  **(*n* = 114)** | | |
| --- | --- | --- | --- | --- | --- | --- | --- | --- | --- | --- | --- | --- | --- | --- | --- | --- |
| **Main category** | **Subcategory** | **Code** | ***k*** | ***%*** | ***k*** | ***%*** | ***c*** | ***k*** | ***%*** | ***c*** | ***k*** | ***%*** | ***c*** | ***k*** | ***%*** | ***c*** |
| Appearance | Athletic |  | 5 | 0.29 | 0 | 0 |  | 1 | 20 |  | 4 | 80 |  | 0 | 0 |  |
|  |  | Athletic body | 4 | 0.23 | 0 | 0 | 0 | 1 | 25 | 0.01 | 3 | 75 | 0.03 | 0 | 0 | 0 |
|  |  | Non-athletic body | 1 | 0.06 | 0 | 0 | 0 | 0 | 0 | 0 | 1 | 100 | 0.01 | 0 | 0 | 0 |
|  | Attractiveness |  | 28 | 1.61 | 9 | 32.14 |  | 5 | 17.86 |  | 6 | 21.43 |  | 8 | 28.57 |  |
|  |  | Attractive | 11 | 0.63 | 5 | 45.45 | 0.04 | 0 | 0 | 0 | 3 | 27.27 | 0.03 | 3 | 27.27 | 0.02 |
|  |  | Attractive face | 4 | 0.23 | 0 | 0 | 0 | 0 | 0 | 0 | 1 | 25 | 0.01 | 3 | 75 | 0.03 |
|  |  | Non-attractive | 12 | 0.69 | 3 | 25 | 0.02 | 5 | 41.67 | 0.04 | 2 | 16.67 | 0.02 | 2 | 16.67 | 0.02 |
|  |  | Standard face | 1 | 0.06 | 1 | 100 | 0.01 | 0 | 0 | 0 | 0 | 0 | 0 | 0 | 0 | 0 |
|  | Care |  | 9 | 0.52 | 4 | 44.44 |  | 2 | 22.22 |  | 2 | 22.22 |  | 1 | 11.11 |  |
|  |  | Care about appearance | 2 | 0.11 | 1 | 50 | 0.01 | 1 | 50 | 0.01 | 0 | 0 | 0 | 0 | 0 | 0 |
|  |  | Do not care about appearance | 7 | 0.4 | 3 | 42.86 | 0.03 | 1 | 14.29 | 0.01 | 2 | 28.57 | 0.02 | 1 | 14.29 | 0.01 |
|  | Clothes |  | 36 | 2.06 | 6 | 16.67 |  | 11 | 30.56 |  | 11 | 30.56 |  | 8 | 22.22 |  |
|  |  | Basic-colored style | 4 | 0.23 | 1 | 25 | 0.01 | 2 | 50 | 0.02 | 0 | 0 | 0 | 1 | 25 | 0.01 |
|  |  | Casual style | 4 | 0.23 | 0 | 0 | 0 | 2 | 50 | 0.02 | 1 | 25 | 0.01 | 1 | 25 | 0.01 |
|  |  | Colored | 3 | 0.17 | 2 | 66.67 | 0.02 | 1 | 33.33 | 0.01 | 0 | 0 | 0 | 0 | 0 | 0 |
|  |  | Dress carelessly | 2 | 0.11 | 0 | 0 | 0 | 0 | 0 | 0 | 0 | 0 | 0 | 2 | 100 | 0.02 |
|  |  | Formal | 14 | 0.8 | 2 | 14.29 | 0.02 | 4 | 28.57 | 0.03 | 6 | 42.86 | 0.05 | 2 | 14.29 | 0.02 |
|  |  | Non-sport | 1 | 0.06 | 0 | 0 | 0 | 0 | 0 | 0 | 1 | 100 | 0.01 | 0 | 0 | 0 |
|  |  | Old-fashioned | 3 | 0.17 | 0 | 0 | 0 | 0 | 0 | 0 | 2 | 66.67 | 0.02 | 1 | 33.33 | 0.01 |
|  |  | S/he wears as s/he wants | 1 | 0.06 | 1 | 100 | 0.01 | 0 | 0 | 0 | 0 | 0 | 0 | 0 | 0 | 0 |
|  |  | Sporty | 4 | 0.23 | 0 | 0 | 0 | 2 | 50 | 0.02 | 1 | 25 | 0.01 | 1 | 25 | 0.01 |
|  | Eyes |  | 34 | 1.95 | 9 | 26.47 |  | 13 | 38.24 |  | 6 | 17.65 |  | 6 | 17.65 |  |
|  |  | Big eyes | 2 | 0.11 | 0 | 0 | 0 | 2 | 100 | 0.02 | 0 | 0 | 0 | 0 | 0 | 0 |
|  |  | Brown/dark eyes | 16 | 0.92 | 4 | 25 | 0.03 | 5 | 31.25 | 0.04 | 4 | 25 | 0.03 | 3 | 18.75 | 0.02 |
|  |  | Light eyes | 15 | 0.86 | 5 | 33.33 | 0.04 | 5 | 33.33 | 0.04 | 2 | 13.33 | 0.02 | 3 | 20 | 0.02 |
|  |  | Small eyes | 1 | 0.06 | 0 | 0 | 0 | 1 | 100 | 0.01 | 0 | 0 | 0 | 0 | 0 | 0 |
|  | Face |  | 179 | 10.3 | 44 | 24.58 |  | 47 | 26.26 |  | 46 | 25.7 |  | 42 | 23.46 |  |
|  |  | Big facial features | 1 | 0.06 | 0 | 0 | 0 | 1 | 100 | 0.01 | 0 | 0 | 0 | 0 | 0 | 0 |
|  |  | Blushing | 1 | 0.06 | 0 | 0 | 0 | 1 | 100 | 0.01 | 0 | 0 | 0 | 0 | 0 | 0 |
|  |  | Bushy eyebrows | 1 | 0.06 | 1 | 100 | 0.01 | 0 | 0 | 0 | 0 | 0 | 0 | 0 | 0 | 0 |
|  |  | Dental braces | 3 | 0.17 | 1 | 33.33 | 0.01 | 1 | 33.33 | 0.01 | 0 | 0 | 0 | 1 | 33.33 | 0.01 |
|  |  | Expressive face | 1 | 0.06 | 1 | 100 | 0.01 | 0 | 0 | 0 | 0 | 0 | 0 | 0 | 0 | 0 |
|  |  | Freckles | 4 | 0.23 | 3 | 75 | 0.03 | 1 | 25 | 0.01 | 0 | 0 | 0 | 0 | 0 | 0 |
|  |  | Grave facial expression | 2 | 0.11 | 2 | 100 | 0.02 | 0 | 0 | 0 | 0 | 0 | 0 | 0 | 0 | 0 |
|  |  | Not very expressive | 1 | 0.06 | 1 | 100 | 0.01 | 0 | 0 | 0 | 0 | 0 | 0 | 0 | 0 | 0 |
|  |  | Not wearing glasses | 4 | 0.23 | 1 | 25 | 0.01 | 1 | 25 | 0.01 | 2 | 50 | 0.02 | 0 | 0 | 0 |
|  |  | Pubertal acne | 4 | 0.23 | 1 | 25 | 0.01 | 1 | 25 | 0.01 | 0 | 0 | 0 | 2 | 50 | 0.02 |
|  |  | Round face | 4 | 0.23 | 2 | 50 | 0.02 | 0 | 0 | 0 | 0 | 0 | 0 | 2 | 50 | 0.02 |
|  |  | Wearing glasses | 153 | 8.77 | 31 | 20.26 | 0.13 | 41 | 26.8 | 0.18 | 44 | 28.76 | 0.2 | 37 | 24.18 | 0.16 |
|  | Hair |  | 221 | 12.7 | 63 | 28.51 |  | 65 | 29.41 |  | 52 | 23.53 |  | 41 | 18.55 |  |
|  |  | Blonde hair | 29 | 1.66 | 10 | 34.48 | 0.08 | 10 | 34.48 | 0.08 | 7 | 24.14 | 0.05 | 2 | 6.897 | 0.01 |
|  |  | Combed hair | 2 | 0.11 | 0 | 0 | 0 | 0 | 0 | 0 | 1 | 50 | 0.01 | 1 | 50 | 0.01 |
|  |  | Curly hair | 13 | 0.75 | 3 | 23.08 | 0.02 | 5 | 38.46 | 0.04 | 4 | 30.77 | 0.03 | 1 | 7.692 | 0.01 |
|  |  | Dark hair | 108 | 6.19 | 28 | 25.93 | 0.14 | 22 | 20.37 | 0.11 | 29 | 26.85 | 0.15 | 29 | 26.85 | 0.15 |
|  |  | Disheveled hair | 4 | 0.23 | 0 | 0 | 0 | 1 | 25 | 0.01 | 1 | 25 | 0.01 | 2 | 50 | 0.02 |
|  |  | Fringe | 3 | 0.17 | 0 | 0 | 0 | 3 | 100 | 0.03 | 0 | 0 | 0 | 0 | 0 | 0 |
|  |  | Ginger hair | 1 | 0.06 | 1 | 100 | 0.01 | 0 | 0 | 0 | 0 | 0 | 0 | 0 | 0 | 0 |
|  |  | Long hair | 13 | 0.75 | 3 | 23.08 | 0.02 | 2 | 15.38 | 0.02 | 5 | 38.46 | 0.04 | 3 | 23.08 | 0.02 |
|  |  | Ponytail/Braids/Headband | 16 | 0.92 | 8 | 50 | 0.07 | 8 | 50 | 0.07 | 0 | 0 | 0 | 0 | 0 | 0 |
|  |  | Short hair | 17 | 0.97 | 3 | 17.65 | 0.02 | 7 | 41.18 | 0.06 | 4 | 23.53 | 0.03 | 3 | 17.65 | 0.02 |
|  |  | Shoulder-length hair | 8 | 0.46 | 5 | 62.5 | 0.04 | 3 | 37.5 | 0.03 | 0 | 0 | 0 | 0 | 0 | 0 |
|  |  | Straight hair | 7 | 0.4 | 2 | 28.57 | 0.02 | 4 | 57.14 | 0.03 | 1 | 14.29 | 0.01 | 0 | 0 | 0 |
|  | Height |  | 229 | 13.1 | 61 | 26.64 |  | 50 | 21.83 |  | 55 | 24.02 |  | 63 | 27.51 |  |
|  |  | Short | 79 | 4.53 | 16 | 20.25 | 0.09 | 26 | 32.91 | 0.16 | 14 | 17.72 | 0.08 | 23 | 29.11 | 0.14 |
|  |  | Standard | 64 | 3.67 | 14 | 21.88 | 0.09 | 13 | 20.31 | 0.08 | 15 | 23.44 | 0.09 | 22 | 34.38 | 0.14 |
|  |  | Tall | 86 | 4.93 | 31 | 36.05 | 0.18 | 11 | 12.79 | 0.06 | 26 | 30.23 | 0.15 | 18 | 20.93 | 0.1 |
|  | Others |  | 48 | 2.75 | 13 | 27.08 |  | 13 | 27.08 |  | 13 | 27.08 |  | 9 | 18.75 |  |
|  |  | Down-headed | 4 | 0.23 | 0 | 0 | 0 | 1 | 25 | 0.01 | 1 | 25 | 0.01 | 2 | 50 | 0.02 |
|  |  | European | 1 | 0.06 | 0 | 0 | 0 | 0 | 0 | 0 | 0 | 0 | 0 | 1 | 100 | 0.01 |
|  |  | Gangly | 2 | 0.11 | 0 | 0 | 0 | 1 | 50 | 0.01 | 0 | 0 | 0 | 1 | 50 | 0.01 |
|  |  | Intellectual appearance | 1 | 0.06 | 1 | 100 | 0.01 | 0 | 0 | 0 | 0 | 0 | 0 | 0 | 0 | 0 |
|  |  | Non-normative physical appearance | 1 | 0.06 | 0 | 0 | 0 | 1 | 100 | 0.01 | 0 | 0 | 0 | 0 | 0 | 0 |
|  |  | Standard global appearance | 38 | 2.18 | 11 | 28.95 | 0.08 | 10 | 26.32 | 0.07 | 12 | 31.58 | 0.09 | 5 | 13.16 | 0.03 |
|  |  | Weak voice | 1 | 0.06 | 1 | 100 | 0.01 | 0 | 0 | 0 | 0 | 0 | 0 | 0 | 0 | 0 |
|  | Physical development |  | 6 | 0.34 | 1 | 16.67 |  | 3 | 50 |  | 1 | 16.67 |  | 1 | 16.67 |  |
|  |  | Age-Preadolescent | 1 | 0.06 | 1 | 100 | 0.01 | 0 | 0 | 0 | 0 | 0 | 0 | 0 | 0 | 0 |
|  |  | Age-Young | 2 | 0.11 | 0 | 0 | 0 | 2 | 100 | 0.02 | 0 | 0 | 0 | 0 | 0 | 0 |
|  |  | Late developed | 3 | 0.17 | 0 | 0 | 0 | 1 | 33.33 | 0.01 | 1 | 33.33 | 0.01 | 1 | 33.33 | 0.01 |
|  | Size |  | 185 | 10.6 | 47 | 25.41 |  | 37 | 20 |  | 45 | 24.32 |  | 56 | 30.27 |  |
|  |  | Overweight | 16 | 0.92 | 3 | 18.75 | 0.02 | 4 | 25 | 0.03 | 2 | 12.5 | 0.02 | 7 | 43.75 | 0.06 |
|  |  | Slender | 5 | 0.29 | 4 | 80 | 0.03 | 1 | 20 | 0.01 | 0 | 0 | 0 | 0 | 0 | 0 |
|  |  | Slim | 118 | 6.77 | 28 | 23.73 | 0.14 | 19 | 16.1 | 0.09 | 31 | 26.27 | 0.16 | 40 | 33.9 | 0.21 |
|  |  | Standard body size | 42 | 2.41 | 11 | 26.19 | 0.08 | 12 | 28.57 | 0.08 | 10 | 23.81 | 0.07 | 9 | 21.43 | 0.06 |
|  |  | Stocky | 4 | 0.23 | 1 | 25 | 0.01 | 1 | 25 | 0.01 | 2 | 50 | 0.02 | 0 | 0 | 0 |
|  | Skin |  | 18 | 1.03 | 6 | 33.33 |  | 5 | 27.78 |  | 4 | 22.22 |  | 3 | 16.67 |  |
|  |  | Pale skin | 13 | 0.75 | 5 | 38.46 | 0.04 | 3 | 23.08 | 0.02 | 3 | 23.08 | 0.02 | 2 | 15.38 | 0.02 |
|  |  | Tanned skin | 5 | 0.29 | 1 | 20 | 0.01 | 2 | 40 | 0.02 | 1 | 20 | 0.01 | 1 | 20 | 0.01 |
| Athletic competence | Athletic competence |  | 45 | 2.58 | 10 | 22.22 |  | 7 | 15.56 |  | 11 | 24.44 |  | 17 | 37.78 |  |
|  |  | Clumsy | 10 | 0.57 | 1 | 10 | 0.01 | 3 | 30 | 0.03 | 1 | 10 | 0.01 | 5 | 50 | 0.04 |
|  |  | Good athletics abilities | 12 | 0.69 | 4 | 33.33 | 0.03 | 2 | 16.67 | 0.02 | 4 | 33.33 | 0.03 | 2 | 16.67 | 0.02 |
|  |  | Not good at sports | 14 | 0.8 | 4 | 28.57 | 0.03 | 2 | 14.29 | 0.02 | 2 | 14.29 | 0.02 | 6 | 42.86 | 0.05 |
|  |  | Standard athletic abilities | 9 | 0.52 | 1 | 11.11 | 0.01 | 0 | 0 | 0 | 4 | 44.44 | 0.03 | 4 | 44.44 | 0.03 |
| Behavior | Athletics |  | 11 | 0.63 | 3 | 27.27 |  | 0 | 0 |  | 3 | 27.27 |  | 5 | 45.45 |  |
|  |  | Do not play sports | 7 | 0.4 | 2 | 28.57 | 0.02 | 0 | 0 | 0 | 2 | 28.57 | 0.02 | 3 | 42.86 | 0.03 |
|  |  | Obliged to athletics | 1 | 0.06 | 0 | 0 | 0 | 0 | 0 | 0 | 1 | 100 | 0.01 | 0 | 0 | 0 |
|  |  | Play sports | 3 | 0.17 | 1 | 33.33 | 0.01 | 0 | 0 | 0 | 0 | 0 | 0 | 2 | 66.67 | 0.02 |
| Fitness | Fitness |  | 56 | 3.21 | 10 | 17.86 |  | 7 | 12.5 |  | 20 | 35.71 |  | 19 | 33.93 |  |
|  |  | High physical fitness | 17 | 0.97 | 2 | 11.76 | 0.02 | 1 | 5.882 | 0.01 | 11 | 64.71 | 0.09 | 3 | 17.65 | 0.02 |
|  |  | Low physical fitness | 18 | 1.03 | 2 | 11.11 | 0.02 | 3 | 16.67 | 0.02 | 4 | 22.22 | 0.03 | 9 | 50 | 0.07 |
|  |  | Standard physical fitness | 21 | 1.2 | 6 | 28.57 | 0.05 | 3 | 14.29 | 0.02 | 5 | 23.81 | 0.04 | 7 | 33.33 | 0.05 |
| Global trait |  |  |  |  |  |  |  |  |  |  |  |  |  |  |  |  |
|  |  | Standard person | 26 | 1.49 | 3 | 11.54 | 0.02 | 4 | 15.38 | 0.03 | 7 | 26.92 | 0.05 | 12 | 46.15 | 0.09 |
| Health | Health |  | 7 | 0.4 | 0 | 0 |  | 0 | 0 |  | 5 | 71.43 |  | 2 | 28.57 |  |
|  |  | Healthy | 1 | 0.06 | 0 | 0 | 0 | 0 | 0 | 0 | 1 | 100 | 0.01 | 0 | 0 | 0 |
|  |  | Little self-care | 1 | 0.06 | 0 | 0 | 0 | 0 | 0 | 0 | 1 | 100 | 0.01 | 0 | 0 | 0 |
|  |  | Physically weak | 5 | 0.29 | 0 | 0 | 0 | 0 | 0 | 0 | 3 | 60 | 0.03 | 2 | 40 | 0.02 |
| Interests | Sports |  | 13 | 0.75 | 4 | 30.77 |  | 1 | 7.692 |  | 3 | 23.08 |  | 5 | 38.46 |  |
|  |  | Can be interested or not in sports | 1 | 0.06 | 1 | 100 | 0.01 | 0 | 0 | 0 | 0 | 0 | 0 | 0 | 0 | 0 |
|  |  | Don't like/Indifferent to physical activity | 11 | 0.63 | 3 | 27.27 | 0.02 | 1 | 9.091 | 0.01 | 2 | 18.18 | 0.02 | 5 | 45.45 | 0.04 |
|  |  | Preference for e-sports | 1 | 0.06 | 0 | 0 | 0 | 0 | 0 | 0 | 1 | 100 | 0.01 | 0 | 0 | 0 |
| Skills/Ability | Physical |  | 2 | 0.11 | 0 | 0 |  | 2 | 100 |  | 0 | 0 |  | 0 | 0 |  |
|  |  | Disability | 1 | 0.06 | 0 | 0 | 0 | 1 | 100 | 0.01 | 0 | 0 | 0 | 0 | 0 | 0 |
|  |  | No Disability | 1 | 0.06 | 0 | 0 | 0 | 1 | 100 | 0.01 | 0 | 0 | 0 | 0 | 0 | 0 |
| Unknown / Cannot determine |  | Unknown / Cannot determine | 30 | 1.72 | 6 | 20 | 0.04 | 8 | 26.67 | 0.06 | 12 | 40 | 0.09 | 4 | 13.33 | 0.03 |
| *Note*: *k*: frequency of codifications. *c*: co-occurrence coefficient. | | | | | | | | | | | | | | | | |

**Table S5**

*Categories, subcategories and codes with frequency and percentage of mentioned physical attributes by ability and gender*

|  |  |  | **All** | | **All gifted**  **(*n* = 225)** | | | **All non-gifted**  **(*n* = 227)** | | | **All girls**  **(*n* = 226)** | | | **All boys**  **(*n* = 226)** | | |
| --- | --- | --- | --- | --- | --- | --- | --- | --- | --- | --- | --- | --- | --- | --- | --- | --- |
| **Main category** | **Subcategory** | **Code** | ***k*** | ***%*** | ***k*** | ***%*** | ***c*** | ***k*** | ***%*** | ***c*** | ***k*** | ***%*** | ***c*** | ***k*** | ***%*** | ***c*** |
| Appearance | Athletic |  | 5 | 0.29 | 4 | 80 |  | 1 | 20 |  | 1 | 20 |  | 4 | 80 |  |
|  |  | Athletic body | 4 | 0.23 | 3 | 75 | 0.01 | 1 | 25 | 0 | 1 | 25 | 0 | 3 | 75 | 0.01 |
|  |  | Non-athletic body | 1 | 0.06 | 1 | 100 | 0 | 0 | 0 | 0 | 0 | 0 | 0 | 1 | 100 | 0 |
|  | Attractiveness |  | 28 | 1.61 | 15 | 53.57 |  | 13 | 46.43 |  | 14 | 50 |  | 14 | 50 |  |
|  |  | Attractive | 11 | 0.63 | 8 | 72.73 | 0.03 | 3 | 27.27 | 0.01 | 5 | 45.45 | 0.02 | 6 | 54.55 | 0.03 |
|  |  | Attractive face | 4 | 0.23 | 1 | 25 | 0 | 3 | 75 | 0.01 | 0 | 0 | 0 | 4 | 100 | 0.02 |
|  |  | Non-attractive | 12 | 0.69 | 5 | 41.67 | 0.02 | 7 | 58.33 | 0.03 | 8 | 66.67 | 0.03 | 4 | 33.33 | 0.02 |
|  |  | Standard face | 1 | 0.06 | 1 | 100 | 0 | 0 | 0 | 0 | 1 | 100 | 0 | 0 | 0 | 0 |
|  | Care |  | 9 | 0.52 | 6 | 66.67 |  | 3 | 33.33 |  | 6 | 66.67 |  | 3 | 33.33 |  |
|  |  | Care about appearance | 2 | 0.11 | 1 | 50 | 0 | 1 | 50 | 0 | 2 | 100 | 0.01 | 0 | 0 | 0 |
|  |  | Do not care about appearance | 7 | 0.4 | 5 | 71.43 | 0.02 | 2 | 28.57 | 0.01 | 4 | 57.14 | 0.02 | 3 | 42.86 | 0.01 |
|  | Clothes |  | 36 | 2.06 | 17 | 47.22 |  | 19 | 52.78 |  | 17 | 47.22 |  | 19 | 52.78 |  |
|  |  | Basic-colored style | 4 | 0.23 | 1 | 25 | 0 | 3 | 75 | 0.01 | 3 | 75 | 0.01 | 1 | 25 | 0 |
|  |  | Casual style | 4 | 0.23 | 1 | 25 | 0 | 3 | 75 | 0.01 | 2 | 50 | 0.01 | 2 | 50 | 0.01 |
|  |  | Colored | 3 | 0.17 | 2 | 66.67 | 0.01 | 1 | 33.33 | 0 | 3 | 100 | 0.01 | 0 | 0 | 0 |
|  |  | Dress carelessly | 2 | 0.11 | 0 | 0 | 0 | 2 | 100 | 0.01 | 0 | 0 | 0 | 2 | 100 | 0.01 |
|  |  | Formal | 14 | 0.8 | 8 | 57.14 | 0.03 | 6 | 42.86 | 0.03 | 6 | 42.86 | 0.03 | 8 | 57.14 | 0.03 |
|  |  | Non-sport | 1 | 0.06 | 1 | 100 | 0 | 0 | 0 | 0 | 0 | 0 | 0 | 1 | 100 | 0 |
|  |  | Old-fashioned | 3 | 0.17 | 2 | 66.67 | 0.01 | 1 | 33.33 | 0 | 0 | 0 | 0 | 3 | 100 | 0.01 |
|  |  | S/he wears as s/he wants | 1 | 0.06 | 1 | 100 | 0 | 0 | 0 | 0 | 1 | 100 | 0 | 0 | 0 | 0 |
|  |  | Sporty | 4 | 0.23 | 1 | 25 | 0 | 3 | 75 | 0.01 | 2 | 50 | 0.01 | 2 | 50 | 0.01 |
|  | Eyes |  | 34 | 1.95 | 15 | 44.12 |  | 19 | 55.88 |  | 22 | 64.71 |  | 12 | 35.29 |  |
|  |  | Big eyes | 2 | 0.11 | 0 | 0 | 0 | 2 | 100 | 0.01 | 2 | 100 | 0.01 | 0 | 0 | 0 |
|  |  | Brown/dark eyes | 16 | 0.92 | 8 | 50 | 0.03 | 8 | 50 | 0.03 | 9 | 56.25 | 0.04 | 7 | 43.75 | 0.03 |
|  |  | Light eyes | 15 | 0.86 | 7 | 46.67 | 0.03 | 8 | 53.33 | 0.03 | 10 | 66.67 | 0.04 | 5 | 33.33 | 0.02 |
|  |  | Small eyes | 1 | 0.06 | 0 | 0 | 0 | 1 | 100 | 0 | 1 | 100 | 0 | 0 | 0 | 0 |
|  | Face |  | 179 | 10.3 | 90 | 50.28 |  | 89 | 49.72 |  | 91 | 50.84 |  | 88 | 49.16 |  |
|  |  | Big facial features | 1 | 0.06 | 0 | 0 | 0 | 1 | 100 | 0 | 1 | 100 | 0 | 0 | 0 | 0 |
|  |  | Blushing | 1 | 0.06 | 0 | 0 | 0 | 1 | 100 | 0 | 1 | 100 | 0 | 0 | 0 | 0 |
|  |  | Bushy eyebrows | 1 | 0.06 | 1 | 100 | 0 | 0 | 0 | 0 | 1 | 100 | 0 | 0 | 0 | 0 |
|  |  | Dental braces | 3 | 0.17 | 1 | 33.33 | 0 | 2 | 66.67 | 0.01 | 2 | 66.67 | 0.01 | 1 | 33.33 | 0 |
|  |  | Expressive face | 1 | 0.06 | 1 | 100 | 0 | 0 | 0 | 0 | 1 | 100 | 0 | 0 | 0 | 0 |
|  |  | Freckles | 4 | 0.23 | 3 | 75 | 0.01 | 1 | 25 | 0 | 4 | 100 | 0.02 | 0 | 0 | 0 |
|  |  | Grave facial expression | 2 | 0.11 | 2 | 100 | 0.01 | 0 | 0 | 0 | 2 | 100 | 0.01 | 0 | 0 | 0 |
|  |  | Not very expressive | 1 | 0.06 | 1 | 100 | 0 | 0 | 0 | 0 | 1 | 100 | 0 | 0 | 0 | 0 |
|  |  | Not wearing glasses | 4 | 0.23 | 3 | 75 | 0.01 | 1 | 25 | 0 | 2 | 50 | 0.01 | 2 | 50 | 0.01 |
|  |  | Pubertal acne | 4 | 0.23 | 1 | 25 | 0 | 3 | 75 | 0.01 | 2 | 50 | 0.01 | 2 | 50 | 0.01 |
|  |  | Round face | 4 | 0.23 | 2 | 50 | 0.01 | 2 | 50 | 0.01 | 2 | 50 | 0.01 | 2 | 50 | 0.01 |
|  |  | Wearing glasses | 153 | 8.77 | 75 | 49.02 | 0.25 | 78 | 50.98 | 0.26 | 72 | 47.06 | 0.23 | 81 | 52.94 | 0.27 |
|  | Hair |  | 221 | 12.7 | 115 | 52.04 |  | 106 | 47.96 |  | 128 | 57.92 |  | 93 | 42.08 |  |
|  |  | Blonde hair | 29 | 1.66 | 17 | 58.62 | 0.07 | 12 | 41.38 | 0.05 | 20 | 68.97 | 0.08 | 9 | 31.03 | 0.04 |
|  |  | Combed hair | 2 | 0.11 | 1 | 50 | 0 | 1 | 50 | 0 | 0 | 0 | 0 | 2 | 100 | 0.01 |
|  |  | Curly hair | 13 | 0.75 | 7 | 53.85 | 0.03 | 6 | 46.15 | 0.03 | 8 | 61.54 | 0.03 | 5 | 38.46 | 0.02 |
|  |  | Dark hair | 108 | 6.19 | 57 | 52.78 | 0.21 | 51 | 47.22 | 0.18 | 50 | 46.3 | 0.18 | 58 | 53.7 | 0.21 |
|  |  | Disheveled hair | 4 | 0.23 | 1 | 25 | 0 | 3 | 75 | 0.01 | 1 | 25 | 0 | 3 | 75 | 0.01 |
|  |  | Fringe | 3 | 0.17 | 0 | 0 | 0 | 3 | 100 | 0.01 | 3 | 100 | 0.01 | 0 | 0 | 0 |
|  |  | Ginger hair | 1 | 0.06 | 1 | 100 | 0 | 0 | 0 | 0 | 1 | 100 | 0 | 0 | 0 | 0 |
|  |  | Long hair | 13 | 0.75 | 8 | 61.54 | 0.03 | 5 | 38.46 | 0.02 | 5 | 38.46 | 0.02 | 8 | 61.54 | 0.03 |
|  |  | Ponytail/Braids/Headband | 16 | 0.92 | 8 | 50 | 0.03 | 8 | 50 | 0.03 | 16 | 100 | 0.07 | 0 | 0 | 0 |
|  |  | Short hair | 17 | 0.97 | 7 | 41.18 | 0.03 | 10 | 58.82 | 0.04 | 10 | 58.82 | 0.04 | 7 | 41.18 | 0.03 |
|  |  | Shoulder-length hair | 8 | 0.46 | 5 | 62.5 | 0.02 | 3 | 37.5 | 0.01 | 8 | 100 | 0.04 | 0 | 0 | 0 |
|  |  | Straight hair | 7 | 0.4 | 3 | 42.86 | 0.01 | 4 | 57.14 | 0.02 | 6 | 85.71 | 0.03 | 1 | 14.29 | 0 |
|  | Height |  | 229 | 13.1 | 116 | 50.66 |  | 113 | 49.34 |  | 111 | 48.47 |  | 118 | 51.53 |  |
|  |  | Short | 79 | 4.53 | 30 | 37.97 | 0.11 | 49 | 62.03 | 0.19 | 42 | 53.16 | 0.16 | 37 | 46.84 | 0.14 |
|  |  | Standard | 64 | 3.67 | 29 | 45.31 | 0.11 | 35 | 54.69 | 0.14 | 27 | 42.19 | 0.1 | 37 | 57.81 | 0.15 |
|  |  | Tall | 86 | 4.93 | 57 | 66.28 | 0.22 | 29 | 33.72 | 0.1 | 42 | 48.84 | 0.15 | 44 | 51.16 | 0.16 |
|  | Others |  | 48 | 2.75 | 26 | 54.17 |  | 22 | 45.83 |  | 26 | 54.17 |  | 22 | 45.83 |  |
|  |  | Down headed | 4 | 0.23 | 1 | 25 | 0 | 3 | 75 | 0.01 | 1 | 25 | 0 | 3 | 75 | 0.01 |
|  |  | European | 1 | 0.06 | 0 | 0 | 0 | 1 | 100 | 0 | 0 | 0 | 0 | 1 | 100 | 0 |
|  |  | Gangly | 2 | 0.11 | 0 | 0 | 0 | 2 | 100 | 0.01 | 1 | 50 | 0 | 1 | 50 | 0 |
|  |  | Intellectual appearance | 1 | 0.06 | 1 | 100 | 0 | 0 | 0 | 0 | 1 | 100 | 0 | 0 | 0 | 0 |
|  |  | Non-normative physical appearance | 1 | 0.06 | 0 | 0 | 0 | 1 | 100 | 0 | 1 | 100 | 0 | 0 | 0 | 0 |
|  |  | Standard global appearance | 38 | 2.18 | 23 | 60.53 | 0.1 | 15 | 39.47 | 0.06 | 21 | 55.26 | 0.09 | 17 | 44.74 | 0.07 |
|  |  | Weak voice | 1 | 0.06 | 1 | 100 | 0 | 0 | 0 | 0 | 1 | 100 | 0 | 0 | 0 | 0 |
|  | Physical development |  | 6 | 0.34 | 2 | 33.33 |  | 4 | 66.67 |  | 4 | 66.67 |  | 2 | 33.33 |  |
|  |  | Age-Preadolescent | 1 | 0.06 | 1 | 100 | 0 | 0 | 0 | 0 | 1 | 100 | 0 | 0 | 0 | 0 |
|  |  | Age-Young | 2 | 0.11 | 0 | 0 | 0 | 2 | 100 | 0.01 | 2 | 100 | 0.01 | 0 | 0 | 0 |
|  |  | Late developed | 3 | 0.17 | 1 | 33.33 | 0 | 2 | 66.67 | 0.01 | 1 | 33.33 | 0 | 2 | 66.67 | 0.01 |
|  | Size |  | 185 | 10.6 | 92 | 49.73 |  | 93 | 50.27 |  | 84 | 45.41 |  | 101 | 54.59 |  |
|  |  | Overweight | 16 | 0.92 | 5 | 31.25 | 0.02 | 11 | 68.75 | 0.05 | 7 | 43.75 | 0.03 | 9 | 56.25 | 0.04 |
|  |  | Slender | 5 | 0.29 | 4 | 80 | 0.02 | 1 | 20 | 0 | 5 | 100 | 0.02 | 0 | 0 | 0 |
|  |  | Slim | 118 | 6.77 | 59 | 50 | 0.21 | 59 | 50 | 0.21 | 47 | 39.83 | 0.16 | 71 | 60.17 | 0.26 |
|  |  | Standard body size | 42 | 2.41 | 21 | 50 | 0.09 | 21 | 50 | 0.08 | 23 | 54.76 | 0.09 | 19 | 45.24 | 0.08 |
|  |  | Stocky | 4 | 0.23 | 3 | 75 | 0.01 | 1 | 25 | 0 | 2 | 50 | 0.01 | 2 | 50 | 0.01 |
|  | Skin |  | 18 | 1.03 | 10 | 55.56 |  | 8 | 44.44 |  | 11 | 61.11 |  | 7 | 38.89 |  |
|  |  | Pale skin | 13 | 0.75 | 8 | 61.54 | 0.03 | 5 | 38.46 | 0.02 | 8 | 61.54 | 0.03 | 5 | 38.46 | 0.02 |
|  |  | Tanned skin | 5 | 0.29 | 2 | 40 | 0.01 | 3 | 60 | 0.01 | 3 | 60 | 0.01 | 2 | 40 | 0.01 |
| Athletic competence | Athletic competence |  | 45 | 2.58 | 21 | 46.67 |  | 24 | 53.33 |  | 17 | 37.78 |  | 28 | 62.22 |  |
|  |  | Clumsy | 10 | 0.57 | 2 | 20.00 | 0.01 | 8 | 80.00 | 0.03 | 4 | 40 | 0.02 | 6 | 60 | 0.03 |
|  |  | Good athletics abilities | 12 | 0.69 | 8 | 66.67 | 0.03 | 4 | 33.33 | 0.02 | 6 | 50 | 0.03 | 6 | 50 | 0.03 |
|  |  | Not good at sports | 14 | 0.8 | 6 | 42.86 | 0.03 | 8 | 57.14 | 0.03 | 6 | 42.86 | 0.03 | 8 | 57.14 | 0.03 |
|  |  | Standard athletic abilities | 9 | 0.52 | 5 | 55.56 | 0.02 | 4 | 44.44 | 0.02 | 1 | 11.11 | 0 | 8 | 88.89 | 0.04 |
| Behavior | Athletics |  | 11 | 0.63 | 6 | 54.55 |  | 5 | 45.45 |  | 3 | 27.27 |  | 8 | 72.73 |  |
|  |  | Do not play sports | 7 | 0.4 | 4 | 57.14 | 0.02 | 3 | 42.86 | 0.01 | 2 | 28.57 | 0.01 | 5 | 71.43 | 0.02 |
|  |  | Obliged to athletics | 1 | 0.06 | 1 | 100 | 0 | 0 | 0 | 0 | 0 | 0 | 0 | 1 | 100 | 0 |
|  |  | Play sports | 3 | 0.17 | 1 | 33.33 | 0 | 2 | 66.67 | 0.01 | 1 | 33.33 | 0 | 2 | 66.67 | 0.01 |
| Fitness | Fitness |  | 56 | 3.21 | 30 | 53.57 |  | 26 | 46.43 |  | 17 | 30.36 |  | 39 | 69.64 |  |
|  |  | High physical fitness | 17 | 0.97 | 13 | 76.47 | 0.06 | 4 | 23.53 | 0.02 | 3 | 17.65 | 0.01 | 14 | 82.35 | 0.06 |
|  |  | Low physical fitness | 18 | 1.03 | 6 | 33.33 | 0.03 | 12 | 66.67 | 0.05 | 5 | 27.78 | 0.02 | 13 | 72.22 | 0.06 |
|  |  | Standard physical fitness | 21 | 1.2 | 11 | 52.38 | 0.05 | 10 | 47.62 | 0.04 | 9 | 42.86 | 0.04 | 12 | 57.14 | 0.05 |
| Global trait |  |  |  |  |  |  |  |  |  |  |  |  |  |  |  |  |
|  |  | Standard person | 26 | 1.49 | 10 | 38.46 | 0.04 | 16 | 61.54 | 0.07 | 7 | 26.92 | 0.03 | 19 | 73.08 | 0.08 |
| Health | Health |  | 7 | 0.4 | 5 | 71.43 |  | 2 | 28.57 |  | 0 | 0 |  | 7 | 100 |  |
|  |  | Healthy | 1 | 0.06 | 1 | 100 | 0 | 0 | 0 | 0 | 0 | 0 | 0 | 1 | 100 | 0 |
|  |  | Little self-care | 1 | 0.06 | 1 | 100 | 0 | 0 | 0 | 0 | 0 | 0 | 0 | 1 | 100 | 0 |
|  |  | Physically weak | 5 | 0.29 | 3 | 60 | 0.01 | 2 | 40 | 0.01 | 0 | 0 | 0 | 5 | 100 | 0.02 |
| Interests | Sports |  | 13 | 0.75 | 7 | 53.85 |  | 6 | 46.15 |  | 5 | 38.46 |  | 8 | 61.54 |  |
|  |  | Can be interested or not in sports | 1 | 0.06 | 1 | 100 | 0 | 0 | 0 | 0 | 1 | 100 | 0 | 0 | 0 | 0 |
|  |  | Don't like/Indifferent to physical activity | 11 | 0.63 | 5 | 45.45 | 0.02 | 6 | 54.55 | 0.03 | 4 | 36.36 | 0.02 | 7 | 63.64 | 0.03 |
|  |  | Preference for e-sports | 1 | 0.06 | 1 | 100 | 0 | 0 | 0 | 0 | 0 | 0 | 0 | 1 | 100 | 0 |
| Skills/Ability | Physical |  | 2 | 0.11 | 0 | 0 |  | 2 | 100 |  | 2 | 100 |  | 0 | 0 |  |
|  |  | Disability | 1 | 0.06 | 0 | 0 | 0 | 1 | 100 | 0 | 1 | 100 | 0 | 0 | 0 | 0 |
|  |  | No Disability | 1 | 0.06 | 0 | 0 | 0 | 1 | 100 | 0 | 1 | 100 | 0 | 0 | 0 | 0 |
| Unknown / Cannot determine |  | Unknown / Cannot determine | 30 | 1.72 | 18 | 60 | 0.08 | 12 | 40 | 0.05 | 14 | 46.67 | 0.06 | 16 | 53.33 | 0.07 |
| *Note*: *k*: frequency of codifications. *c*: co-occurrence coefficient. | | | | | | | | | | | | | | | | |

**Table S6**

*Categories, subcategories and codes with frequency and percentage of mentioned non-physical attributes by vignette*

|  |  |  | **All** | | **All Gifted**  **(*n* = 225)** | | | **All non-gifted**  **(*n* = 227)** | | | **All girls**  **(*n* = 226)** | | | **All boys**  **(*n* = 226)** | | |
| --- | --- | --- | --- | --- | --- | --- | --- | --- | --- | --- | --- | --- | --- | --- | --- | --- |
| **Main category** | **Subcategory** | **Code** | ***k*** | ***%*** | ***k*** | ***%*** | ***c*** | ***k*** | ***%*** | ***c*** | ***k*** | ***%*** | ***c*** | ***k*** | ***%*** | ***c*** |
| Behavior | Academics |  | 24 | 1.38 | 12 | 50.00 |  | 12 | 50.00 |  | 13 | 54.17 |  | 11 | 45.83 |  |
|  |  | Active and participative in class | 2 | 0.11 | 2 | 100.00 | 0.01 | 0 | 0.00 | 0 | 1 | 50.00 | 0 | 1 | 50.00 | 0 |
|  |  | Asks questions | 3 | 0.17 | 1 | 33.33 | 0 | 2 | 66.67 | 0.01 | 1 | 33.33 | 0 | 2 | 66.67 | 0.01 |
|  |  | Get bored in class | 3 | 0.17 | 2 | 66.67 | 0.01 | 1 | 33.33 | 0 | 2 | 66.67 | 0.01 | 1 | 33.33 | 0 |
|  |  | Hard worker | 1 | 0.06 | 1 | 100.00 | 0 | 0 | 0.00 | 0 | 0 | 0.00 | 0 | 1 | 100.00 | 0 |
|  |  | Not disruptive in class | 1 | 0.06 | 1 | 100.00 | 0 | 0 | 0.00 | 0 | 1 | 100.00 | 0 | 0 | 0.00 | 0 |
|  |  | Not possibility to learn more | 1 | 0.06 | 1 | 100.00 | 0 | 0 | 0.00 | 0 | 0 | 0.00 | 0 | 1 | 100.00 | 0 |
|  |  | Show knowledge | 4 | 0.23 | 1 | 25.00 | 0 | 3 | 75.00 | 0.01 | 1 | 25.00 | 0 | 3 | 75.00 | 0.01 |
|  |  | Teacher-dependent | 9 | 0.52 | 3 | 33.33 | 0.01 | 6 | 66.67 | 0.03 | 7 | 77.78 | 0.03 | 2 | 22.22 | 0.01 |
|  | Social |  | 13 | 0.75 | 8 | 61.54 |  | 5 | 38.46 |  | 4 | 30.77 |  | 9 | 69.23 |  |
|  |  | Actions alone | 2 | 0.11 | 2 | 100.00 | 0.01 | 0 | 0.00 | 0 | 0 | 0.00 | 0 | 2 | 100.00 | 0.01 |
|  |  | Disruptive | 3 | 0.17 | 1 | 33.33 | 0 | 2 | 66.67 | 0.01 | 0 | 0.00 | 0 | 3 | 100.00 | 0.01 |
|  |  | S/he correct her/his classmates in case of mistake | 1 | 0.06 | 1 | 100.00 | 0 | 0 | 0.00 | 0 | 0 | 0.00 | 0 | 1 | 100.00 | 0 |
|  |  | Well-behaved | 7 | 0.40 | 4 | 57.14 | 0.02 | 3 | 42.86 | 0.01 | 4 | 57.14 | 0.02 | 3 | 42.86 | 0.01 |
| Feelings / Mental states | Feelings / Mental states |  | 6 | 0.34 | 5 | 83.33 |  | 1 | 16.67 |  | 1 | 16.67 |  | 5 | 83.33 |  |
|  |  | Confused | 1 | 0.06 | 1 | 100.00 | 0 | 0 | 0.00 | 0 | 0 | 0.00 | 0 | 1 | 100.00 | 0 |
|  |  | Do not feel heard | 1 | 0.06 | 0 | 0.00 | 0 | 1 | 100.00 | 0 | 0 | 0.00 | 0 | 1 | 100.00 | 0 |
|  |  | Feel different | 3 | 0.17 | 3 | 100.00 | 0.01 | 0 | 0.00 | 0 | 1 | 33.33 | 0 | 2 | 66.67 | 0.01 |
|  |  | Frustrated | 1 | 0.06 | 1 | 100.00 | 0 | 0 | 0.00 | 0 | 0 | 0.00 | 0 | 1 | 100.00 | 0 |
| Interests | Academic / Intellectual | Academically / Intellectually engaged | 28 | 1.61 | 14 | 50.00 | 0.06 | 14 | 50.00 | 0.06 | 15 | 53.57 | 0.06 | 13 | 46.43 | 0.05 |
|  | Global |  | 33 | 1.89 | 12 | 36.36 |  | 21 | 63.64 |  | 12 | 36.36 |  | 21 | 63.64 |  |
|  |  | Different interests from their peers | 8 | 0.46 | 1 | 12.50 | 0 | 7 | 87.50 | 0.03 | 2 | 25.00 | 0.01 | 6 | 75.00 | 0.03 |
|  |  | Like learning | 25 | 1.43 | 11 | 44.00 | 0.05 | 14 | 56.00 | 0.06 | 10 | 40.00 | 0.04 | 15 | 60.00 | 0.06 |
|  | Others |  | 8 | 0.46 | 5 | 62.50 |  | 3 | 37.50 |  | 5 | 62.50 |  | 3 | 37.50 |  |
|  |  | Like animals | 1 | 0.06 | 1 | 100.00 | 0 | 0 | 0.00 | 0 | 1 | 100.00 | 0 | 0 | 0.00 | 0 |
|  |  | Like calm activities | 2 | 0.11 | 1 | 50.00 | 0 | 1 | 50.00 | 0 | 0 | 0.00 | 0 | 2 | 100.00 | 0.01 |
|  |  | Like creativity | 1 | 0.06 | 0 | 0.00 | 0 | 1 | 100.00 | 0 | 1 | 100.00 | 0 | 0 | 0.00 | 0 |
|  |  | Like music | 2 | 0.11 | 1 | 50.00 | 0 | 1 | 50.00 | 0 | 2 | 100.00 | 0.01 | 0 | 0.00 | 0 |
|  |  | Like playing cards | 1 | 0.06 | 1 | 100.00 | 0 | 0 | 0.00 | 0 | 0 | 0.00 | 0 | 1 | 100.00 | 0 |
|  |  | Like touching | 1 | 0.06 | 1 | 100.00 | 0 | 0 | 0.00 | 0 | 1 | 100.00 | 0 | 0 | 0.00 | 0 |
| Personality | Conscientiousness High |  | 23 | 1.32 | 12 | 52.17 |  | 11 | 47.83 |  | 10 | 43.48 |  | 13 | 56.52 |  |
|  | Conscientiousness High | Conscientiousness High (code) | 16 | 0.92 | 9 | 56.25 | 0.04 | 7 | 43.75 | 0.03 | 8 | 50.00 | 0.03 | 8 | 50.00 | 0.03 |
|  |  | Active | 6 | 0.34 | 3 | 50.00 | 0.01 | 3 | 50.00 | 0.01 | 2 | 33.33 | 0.01 | 4 | 66.67 | 0.02 |
|  |  | Focused | 3 | 0.17 | 0 | 0.00 | 0 | 3 | 100.00 | 0.01 | 1 | 33.33 | 0 | 2 | 66.67 | 0.01 |
|  |  | Organized | 2 | 0.11 | 1 | 50.00 | 0 | 1 | 50.00 | 0 | 1 | 50.00 | 0 | 1 | 50.00 | 0 |
|  |  | Reflective | 3 | 0.17 | 3 | 100.00 | 0.01 | 0 | 0.00 | 0 | 1 | 33.33 | 0 | 2 | 66.67 | 0.01 |
|  |  | Responsible | 4 | 0.23 | 2 | 50.00 | 0.01 | 2 | 50.00 | 0.01 | 2 | 50.00 | 0.01 | 2 | 50.00 | 0.01 |
|  |  | Self-confident | 2 | 0.11 | 1 | 50.00 | 0 | 1 | 50.00 | 0 | 2 | 100.00 | 0.01 | 0 | 0.00 | 0 |
|  |  | Striving | 3 | 0.17 | 2 | 66.67 | 0.01 | 1 | 33.33 | 0 | 1 | 33.33 | 0 | 2 | 66.67 | 0.01 |
|  | Conscientiousness Low |  | 2 | 0.11 | 1 | 50.00 |  | 1 | 50.00 |  | 0 | 0.00 |  | 2 | 100.00 |  |
|  |  | Conscientiousness Low (code) | 2 | 0.11 | 1 | 50.00 | 0 | 1 | 50.00 | 0 | 0 | 0.00 | 0 | 2 | 100.00 | 0.01 |
|  |  | Distracted | 1 | 0.06 | 0 | 0.00 | 0 | 1 | 100.00 | 0 | 0 | 0.00 | 0 | 1 | 100.00 | 0 |
|  |  | Passive | 1 | 0.06 | 1 | 100.00 | 0 | 0 | 0.00 | 0 | 0 | 0.00 | 0 | 1 | 100.00 | 0 |
|  | Extraversion High |  | 8 | 0.46 | 5 | 62.50 |  | 3 | 37.50 |  | 6 | 75.00 |  | 2 | 25.00 |  |
|  |  | Extraversion High (code) | 8 | 0.46 | 5 | 62.50 | 0.02 | 3 | 37.50 | 0.01 | 6 | 75.00 | 0.03 | 2 | 25.00 | 0.01 |
|  |  | Extraverted | 4 | 0.23 | 2 | 50.00 | 0.01 | 2 | 50.00 | 0.01 | 3 | 75.00 | 0.01 | 1 | 25.00 | 0 |
|  |  | Sociable | 4 | 0.23 | 3 | 75.00 | 0.01 | 1 | 25.00 | 0 | 3 | 75.00 | 0.01 | 1 | 25.00 | 0 |
|  | Extraversion Low |  | 68 | 3.90 | 28 | 41.18 |  | 40 | 58.82 |  | 33 | 48.53 |  | 35 | 51.47 |  |
|  |  | Extraversion Low (code) | 67 | 3.84 | 27 | 40.30 | 0.1 | 40 | 59.70 | 0.16 | 32 | 47.76 | 0.12 | 35 | 52.24 | 0.14 |
|  |  | Independent | 5 | 0.29 | 4 | 80.00 | 0.02 | 1 | 20.00 | 0 | 4 | 80.00 | 0.02 | 1 | 20.00 | 0 |
|  |  | Introspective | 2 | 0.11 | 1 | 50.00 | 0 | 1 | 50.00 | 0 | 2 | 100.00 | 0.01 | 0 | 0.00 | 0 |
|  |  | Not very sociable | 5 | 0.29 | 3 | 60.00 | 0.01 | 2 | 40.00 | 0.01 | 3 | 60.00 | 0.01 | 2 | 40.00 | 0.01 |
|  |  | Shy/Introverted | 56 | 3.21 | 20 | 35.71 | 0.08 | 36 | 64.29 | 0.15 | 24 | 42.86 | 0.09 | 32 | 57.14 | 0.13 |
|  | Kindness High |  | 59 | 3.38 | 30 | 50.85 |  | 29 | 49.15 |  | 35 | 59.32 |  | 24 | 40.68 |  |
|  | Kindness High | Kindness High (code) | 46 | 2.64 | 22 | 47.83 | 0.09 | 24 | 52.17 | 0.1 | 26 | 56.52 | 0.11 | 20 | 43.48 | 0.08 |
|  |  | Altruism | 2 | 0.11 | 1 | 50.00 | 0 | 1 | 50.00 | 0 | 1 | 50.00 | 0 | 1 | 50.00 | 0 |
|  |  | Empathetic | 4 | 0.23 | 2 | 50.00 | 0.01 | 2 | 50.00 | 0.01 | 2 | 50.00 | 0.01 | 2 | 50.00 | 0.01 |
|  |  | Friendly | 9 | 0.52 | 5 | 55.56 | 0.02 | 4 | 44.44 | 0.02 | 7 | 77.78 | 0.03 | 2 | 22.22 | 0.01 |
|  |  | Helpful | 7 | 0.40 | 4 | 57.14 | 0.02 | 3 | 42.86 | 0.01 | 4 | 57.14 | 0.02 | 3 | 42.86 | 0.01 |
|  |  | Kind | 27 | 1.55 | 12 | 44.44 | 0.05 | 15 | 55.56 | 0.06 | 16 | 59.26 | 0.07 | 11 | 40.74 | 0.05 |
|  |  | Protector | 1 | 0.06 | 0 | 0.00 | 0 | 1 | 100.00 | 0 | 0 | 0.00 | 0 | 1 | 100.00 | 0 |
|  |  | Respectful | 3 | 0.17 | 2 | 66.67 | 0.01 | 1 | 33.33 | 0 | 2 | 66.67 | 0.01 | 1 | 33.33 | 0 |
|  |  | Sensitive | 6 | 0.34 | 4 | 66.67 | 0.02 | 2 | 33.33 | 0.01 | 3 | 50.00 | 0.01 | 3 | 50.00 | 0.01 |
|  | Kindness Low |  | 2 | 0.11 | 2 | 100.00 |  | 0 | 0.00 |  | 0 | 0.00 |  | 2 | 100.00 |  |
|  | Kindness Low | Kindness Low (code) | 1 | 0.06 | 1 | 100.00 | 0 | 0 | 0.00 | 0 | 0 | 0.00 | 0 | 1 | 100.00 | 0 |
|  |  | Antisocial | 1 | 0.06 | 1 | 100.00 | 0 | 0 | 0.00 | 0 | 0 | 0.00 | 0 | 1 | 100.00 | 0 |
|  |  | Apathetic | 1 | 0.06 | 1 | 100.00 | 0 | 0 | 0.00 | 0 | 0 | 0.00 | 0 | 1 | 100.00 | 0 |
|  | Neuroticism High |  | 32 | 1.83 | 14 | 43.75 |  | 18 | 56.25 |  | 15 | 46.88 |  | 17 | 53.13 |  |
|  | Neuroticism High | Neuroticism High (code) | 29 | 1.66 | 12 | 41.38 | 0.05 | 17 | 58.62 | 0.07 | 15 | 51.72 | 0.06 | 14 | 48.28 | 0.06 |
|  |  | Anxious | 1 | 0.06 | 0 | 0.00 | 0 | 1 | 100.00 | 0 | 0 | 0.00 | 0 | 1 | 100.00 | 0 |
|  |  | Compulsive | 1 | 0.06 | 0 | 0.00 | 0 | 1 | 100.00 | 0 | 0 | 0.00 | 0 | 1 | 100.00 | 0 |
|  |  | Dependent | 5 | 0.29 | 0 | 0.00 | 0 | 5 | 100.00 | 0.02 | 5 | 100.00 | 0.02 | 0 | 0.00 | 0 |
|  |  | Emotionally weak | 1 | 0.06 | 1 | 100.00 | 0 | 0 | 0.00 | 0 | 0 | 0.00 | 0 | 1 | 100.00 | 0 |
|  |  | Impatient | 1 | 0.06 | 1 | 100.00 | 0 | 0 | 0.00 | 0 | 0 | 0.00 | 0 | 1 | 100.00 | 0 |
|  |  | Inferiority complex | 2 | 0.11 | 0 | 0.00 | 0 | 2 | 100.00 | 0.01 | 1 | 50.00 | 0 | 1 | 50.00 | 0 |
|  |  | Insecure | 11 | 0.63 | 6 | 54.55 | 0.03 | 5 | 45.45 | 0.02 | 4 | 36.36 | 0.02 | 7 | 63.64 | 0.03 |
|  |  | Maladjusted | 6 | 0.34 | 4 | 66.67 | 0.02 | 2 | 33.33 | 0.01 | 3 | 50.00 | 0.01 | 3 | 50.00 | 0.01 |
|  |  | Mulish | 1 | 0.06 | 1 | 100.00 | 0 | 0 | 0.00 | 0 | 0 | 0.00 | 0 | 1 | 100.00 | 0 |
|  |  | Restless | 2 | 0.11 | 1 | 50.00 | 0 | 1 | 50.00 | 0 | 1 | 50.00 | 0 | 1 | 50.00 | 0 |
|  |  | Temperamental | 1 | 0.06 | 0 | 0.00 | 0 | 1 | 100.00 | 0 | 1 | 100.00 | 0 | 0 | 0.00 | 0 |
|  | Neuroticism Low |  | 3 | 0.17 | 2 | 66.67 |  | 1 | 33.33 |  | 1 | 33.33 |  | 2 | 66.67 |  |
|  | Neuroticism Low | Personality Neuroticism Low (code) | 3 | 0.17 | 2 | 66.67 | 0.01 | 1 | 33.33 | 0 | 1 | 33.33 | 0 | 2 | 66.67 | 0.01 |
|  |  | Calm | 2 | 0.11 | 1 | 50.00 | 0 | 1 | 50.00 | 0 | 0 | 0.00 | 0 | 2 | 100.00 | 0.01 |
|  |  | Patient | 1 | 0.06 | 1 | 100.00 | 0 | 0 | 0.00 | 0 | 1 | 100.00 | 0 | 0 | 0.00 | 0 |
|  | Openness to experience High |  | 53 | 3.04 | 25 | 47.17 |  | 28 | 52.83 |  | 28 | 52.83 |  | 25 | 47.17 |  |
|  | Openness to experience High | Personality Openness to experience High (code) | 49 | 2.81 | 22 | 44.90 | 0.09 | 27 | 55.10 | 0.11 | 26 | 53.06 | 0.1 | 23 | 46.94 | 0.09 |
|  |  | Ambitious | 6 | 0.34 | 4 | 66.67 | 0.02 | 2 | 33.33 | 0.01 | 3 | 50.00 | 0.01 | 3 | 50.00 | 0.01 |
|  |  | Curious | 38 | 2.18 | 15 | 39.47 | 0.06 | 23 | 60.53 | 0.1 | 20 | 52.63 | 0.08 | 18 | 47.37 | 0.07 |
|  |  | Funny | 3 | 0.17 | 1 | 33.33 | 0 | 2 | 66.67 | 0.01 | 2 | 66.67 | 0.01 | 1 | 33.33 | 0 |
|  |  | Imaginative/Intuitive | 3 | 0.17 | 3 | 100.00 | 0.01 | 0 | 0.00 | 0 | 2 | 66.67 | 0.01 | 1 | 33.33 | 0 |
|  |  | Logic | 1 | 0.06 | 1 | 100.00 | 0 | 0 | 0.00 | 0 | 1 | 100.00 | 0 | 0 | 0.00 | 0 |
|  |  | Perceptive | 1 | 0.06 | 0 | 0.00 | 0 | 1 | 100.00 | 0 | 0 | 0.00 | 0 | 1 | 100.00 | 0 |
|  |  | Tolerant | 1 | 0.06 | 1 | 100.00 | 0 | 0 | 0.00 | 0 | 0 | 0.00 | 0 | 1 | 100.00 | 0 |
|  | Openness to experience Low |  | 1 | 0.06 | 0 | 0.00 |  | 1 | 100.00 |  | 1 | 100.00 |  | 0 | 0.00 |  |
|  | Openness to experience Low | Personality Openness to experience Low (code) | 1 | 0.06 | 0 | 0.00 | 0 | 1 | 100.00 | 0 | 1 | 100.00 | 0 | 0 | 0.00 | 0 |
|  |  | Conservatism | 1 | 0.06 | 0 | 0.00 | 0 | 1 | 100.00 | 0 | 1 | 100.00 | 0 | 0 | 0.00 | 0 |
| Self-perceptions | Self-perceptions |  | 6 | 0.34 | 1 | 16.67 |  | 5 | 83.33 |  | 2 | 33.33 |  | 4 | 66.67 |  |
|  |  | Low self-esteem | 5 | 0.29 | 0 | 0.00 | 0 | 5 | 100.00 | 0.02 | 1 | 20.00 | 0 | 4 | 80.00 | 0.02 |
|  |  | Not aware of her/his own attractiveness | 1 | 0.06 | 1 | 100.00 | 0 | 0 | 0.00 | 0 | 1 | 100.00 | 0 | 0 | 0.00 | 0 |
| Skills/Ability | Academic |  | 18 | 1.03 | 9 | 50.00 |  | 9 | 50.00 |  | 6 | 33.33 |  | 12 | 66.67 |  |
|  |  | High academic performance | 11 | 0.63 | 5 | 45.45 | 0.02 | 6 | 54.55 | 0.03 | 3 | 27.27 | 0.01 | 8 | 72.73 | 0.03 |
|  |  | Low academic performance | 4 | 0.23 | 2 | 50.00 | 0.01 | 2 | 50.00 | 0.01 | 2 | 50.00 | 0.01 | 2 | 50.00 | 0.01 |
|  |  | Need help to exploit his/her abilities | 1 | 0.06 | 1 | 100.00 | 0 | 0 | 0.00 | 0 | 1 | 100.00 | 0 | 0 | 0.00 | 0 |
|  |  | No skilled in art activities | 1 | 0.06 | 1 | 100.00 | 0 | 0 | 0.00 | 0 | 0 | 0.00 | 0 | 1 | 100.00 | 0 |
|  |  | Standard ability | 1 | 0.06 | 0 | 0.00 | 0 | 1 | 100.00 | 0 | 0 | 0.00 | 0 | 1 | 100.00 | 0 |
|  | Coping |  | 4 | 0.23 | 2 | 50.00 |  | 2 | 50.00 |  | 1 | 25.00 |  | 3 | 75.00 |  |
|  |  | Ignoring classmates' mockery | 1 | 0.06 | 1 | 100.00 | 0 | 0 | 0.00 | 0 | 0 | 0.00 | 0 | 1 | 100.00 | 0 |
|  |  | Masking | 3 | 0.17 | 1 | 33.33 | 0 | 2 | 66.67 | 0.01 | 1 | 33.33 | 0 | 2 | 66.67 | 0.01 |
|  | Intellectual/cognitive |  | 58 | 3.33 | 37 | 63.79 |  | 21 | 36.21 |  | 34 | 58.62 |  | 24 | 41.38 |  |
|  |  | Able in different knowledge areas | 5 | 0.29 | 5 | 100.00 | 0.02 | 0 | 0.00 | 0 | 2 | 40.00 | 0.01 | 3 | 60.00 | 0.01 |
|  |  | Concentration | 3 | 0.17 | 2 | 66.67 | 0.01 | 1 | 33.33 | 0 | 1 | 33.33 | 0 | 2 | 66.67 | 0.01 |
|  |  | Creative | 2 | 0.11 | 2 | 100.00 | 0.01 | 0 | 0.00 | 0 | 2 | 100.00 | 0.01 | 0 | 0.00 | 0 |
|  |  | Disability | 1 | 0.06 | 0 | 0.00 | 0 | 1 | 100.00 | 0 | 0 | 0.00 | 0 | 1 | 100.00 | 0 |
|  |  | Fast in combining clothes | 1 | 0.06 | 1 | 100.00 | 0 | 0 | 0.00 | 0 | 1 | 100.00 | 0 | 0 | 0.00 | 0 |
|  |  | Intelligent | 44 | 2.52 | 27 | 61.36 | 0.11 | 17 | 38.64 | 0.07 | 26 | 59.09 | 0.11 | 18 | 40.91 | 0.07 |
|  |  | More skilled in theoretical subjects than practical or sports | 1 | 0.06 | 0 | 0.00 | 0 | 1 | 100.00 | 0 | 1 | 100.00 | 0 | 0 | 0.00 | 0 |
|  |  | No Disability | 1 | 0.06 | 0 | 0.00 | 0 | 1 | 100.00 | 0 | 1 | 100.00 | 0 | 0 | 0.00 | 0 |
|  | Socio-emotional |  | 35 | 2.01 | 15 | 42.86 |  | 20 | 57.14 |  | 19 | 54.29 |  | 16 | 45.71 |  |
|  |  | Good at talking | 1 | 0.06 | 0 | 0.00 | 0 | 1 | 100.00 | 0 | 1 | 100.00 | 0 | 0 | 0.00 | 0 |
|  |  | Great emotional intelligence | 1 | 0.06 | 1 | 100.00 | 0 | 0 | 0.00 | 0 | 0 | 0.00 | 0 | 1 | 100.00 | 0 |
|  |  | Low social skills | 31 | 1.78 | 14 | 45.16 | 0.06 | 17 | 54.84 | 0.07 | 17 | 54.84 | 0.07 | 14 | 45.16 | 0.06 |
|  |  | Standard social skills | 2 | 0.11 | 0 | 0.00 | 0 | 2 | 100.00 | 0.01 | 1 | 50.00 | 0 | 1 | 50.00 | 0 |
| Social | Social |  | 35 | 2.01 | 13 | 37.14 |  | 22 | 62.86 |  | 15 | 42.86 |  | 20 | 57.14 |  |
|  |  | Bullying | 2 | 0.11 | 1 | 50.00 | 0 | 1 | 50.00 | 0 | 0 | 0.00 | 0 | 2 | 100.00 | 0.01 |
|  |  | Few friends | 6 | 0.34 | 2 | 33.33 | 0.01 | 4 | 66.67 | 0.02 | 3 | 50.00 | 0.01 | 3 | 50.00 | 0.01 |
|  |  | Freak/Nerd | 7 | 0.40 | 3 | 42.86 | 0.01 | 4 | 57.14 | 0.02 | 0 | 0.00 | 0 | 7 | 100.00 | 0.03 |
|  |  | Many friends | 1 | 0.06 | 0 | 0.00 | 0 | 1 | 100.00 | 0 | 1 | 100.00 | 0 | 0 | 0.00 | 0 |
|  |  | Misunderstood | 2 | 0.11 | 1 | 50.00 | 0 | 1 | 50.00 | 0 | 1 | 50.00 | 0 | 1 | 50.00 | 0 |
|  |  | Not popular | 7 | 0.40 | 2 | 28.57 | 0.01 | 5 | 71.43 | 0.02 | 3 | 42.86 | 0.01 | 4 | 57.14 | 0.02 |
|  |  | Rejected | 10 | 0.57 | 4 | 40.00 | 0.02 | 6 | 60.00 | 0.03 | 7 | 70.00 | 0.03 | 3 | 30.00 | 0.01 |
| *Note*: *k*: frequency of codifications. *c*: co-occurrence coefficient. | | | | | | | | | | | | | | | | |

**Table S7**

*Categories, subcategories and codes with frequency and percentage of mentioned non-physical attributes by ability and gender*

|  |  |  | **All** | | **All Gifted**  **(*n* = 225)** | | | **All non-gifted**  **(*n* = 227)** | | | **All girls**  **(*n* = 226)** | | | **All boys**  **(*n* = 226)** | | |
| --- | --- | --- | --- | --- | --- | --- | --- | --- | --- | --- | --- | --- | --- | --- | --- | --- |
| **Main category** | **Subcategory** | **Code** | ***k*** | ***%*** | ***k*** | ***%*** | ***c*** | ***k*** | ***%*** | ***c*** | ***k*** | ***%*** | ***c*** | ***k*** | ***%*** | ***c*** |
| Behavior | Academics |  | 24 | 1.38 | 12 | 50.00 |  | 12 | 50.00 |  | 13 | 54.17 |  | 11 | 45.83 |  |
|  |  | Active and participative in class | 2 | 0.11 | 2 | 100.00 | 0.01 | 0 | 0.00 | 0 | 1 | 50.00 | 0 | 1 | 50.00 | 0 |
|  |  | Asks questions | 3 | 0.17 | 1 | 33.33 | 0 | 2 | 66.67 | 0.01 | 1 | 33.33 | 0 | 2 | 66.67 | 0.01 |
|  |  | Get bored in class | 3 | 0.17 | 2 | 66.67 | 0.01 | 1 | 33.33 | 0 | 2 | 66.67 | 0.01 | 1 | 33.33 | 0 |
|  |  | Hard worker | 1 | 0.06 | 1 | 100.00 | 0 | 0 | 0.00 | 0 | 0 | 0.00 | 0 | 1 | 100.00 | 0 |
|  |  | Not disruptive in class | 1 | 0.06 | 1 | 100.00 | 0 | 0 | 0.00 | 0 | 1 | 100.00 | 0 | 0 | 0.00 | 0 |
|  |  | Not possibility to learn more | 1 | 0.06 | 1 | 100.00 | 0 | 0 | 0.00 | 0 | 0 | 0.00 | 0 | 1 | 100.00 | 0 |
|  |  | Show knowledge | 4 | 0.23 | 1 | 25.00 | 0 | 3 | 75.00 | 0.01 | 1 | 25.00 | 0 | 3 | 75.00 | 0.01 |
|  |  | Teacher-dependent | 9 | 0.52 | 3 | 33.33 | 0.01 | 6 | 66.67 | 0.03 | 7 | 77.78 | 0.03 | 2 | 22.22 | 0.01 |
|  | Social |  | 13 | 0.75 | 8 | 61.54 |  | 5 | 38.46 |  | 4 | 30.77 |  | 9 | 69.23 |  |
|  |  | Actions alone | 2 | 0.11 | 2 | 100.00 | 0.01 | 0 | 0.00 | 0 | 0 | 0.00 | 0 | 2 | 100.00 | 0.01 |
|  |  | Disruptive | 3 | 0.17 | 1 | 33.33 | 0 | 2 | 66.67 | 0.01 | 0 | 0.00 | 0 | 3 | 100.00 | 0.01 |
|  |  | S/he correct her/his classmates in case of mistake | 1 | 0.06 | 1 | 100.00 | 0 | 0 | 0.00 | 0 | 0 | 0.00 | 0 | 1 | 100.00 | 0 |
|  |  | Well-behaved | 7 | 0.40 | 4 | 57.14 | 0.02 | 3 | 42.86 | 0.01 | 4 | 57.14 | 0.02 | 3 | 42.86 | 0.01 |
| Feelings / Mental states | Feelings / Mental states |  | 6 | 0.34 | 5 | 83.33 |  | 1 | 16.67 |  | 1 | 16.67 |  | 5 | 83.33 |  |
|  |  | Confused | 1 | 0.06 | 1 | 100.00 | 0 | 0 | 0.00 | 0 | 0 | 0.00 | 0 | 1 | 100.00 | 0 |
|  |  | Do not feel heard | 1 | 0.06 | 0 | 0.00 | 0 | 1 | 100.00 | 0 | 0 | 0.00 | 0 | 1 | 100.00 | 0 |
|  |  | Feel different | 3 | 0.17 | 3 | 100.00 | 0.01 | 0 | 0.00 | 0 | 1 | 33.33 | 0 | 2 | 66.67 | 0.01 |
|  |  | Frustrated | 1 | 0.06 | 1 | 100.00 | 0 | 0 | 0.00 | 0 | 0 | 0.00 | 0 | 1 | 100.00 | 0 |
| Interests | Academic / Intellectual | Academically / Intellectually engaged | 28 | 1.61 | 14 | 50.00 | 0.06 | 14 | 50.00 | 0.06 | 15 | 53.57 | 0.06 | 13 | 46.43 | 0.05 |
|  | Global |  | 33 | 1.89 | 12 | 36.36 |  | 21 | 63.64 |  | 12 | 36.36 |  | 21 | 63.64 |  |
|  |  | Different interests from their peers | 8 | 0.46 | 1 | 12.50 | 0 | 7 | 87.50 | 0.03 | 2 | 25.00 | 0.01 | 6 | 75.00 | 0.03 |
|  |  | Like learning | 25 | 1.43 | 11 | 44.00 | 0.05 | 14 | 56.00 | 0.06 | 10 | 40.00 | 0.04 | 15 | 60.00 | 0.06 |
|  | Others |  | 8 | 0.46 | 5 | 62.50 |  | 3 | 37.50 |  | 5 | 62.50 |  | 3 | 37.50 |  |
|  |  | Like animals | 1 | 0.06 | 1 | 100.00 | 0 | 0 | 0.00 | 0 | 1 | 100.00 | 0 | 0 | 0.00 | 0 |
|  |  | Like calm activities | 2 | 0.11 | 1 | 50.00 | 0 | 1 | 50.00 | 0 | 0 | 0.00 | 0 | 2 | 100.00 | 0.01 |
|  |  | Like creativity | 1 | 0.06 | 0 | 0.00 | 0 | 1 | 100.00 | 0 | 1 | 100.00 | 0 | 0 | 0.00 | 0 |
|  |  | Like music | 2 | 0.11 | 1 | 50.00 | 0 | 1 | 50.00 | 0 | 2 | 100.00 | 0.01 | 0 | 0.00 | 0 |
|  |  | Like playing cards | 1 | 0.06 | 1 | 100.00 | 0 | 0 | 0.00 | 0 | 0 | 0.00 | 0 | 1 | 100.00 | 0 |
|  |  | Like touching | 1 | 0.06 | 1 | 100.00 | 0 | 0 | 0.00 | 0 | 1 | 100.00 | 0 | 0 | 0.00 | 0 |
| Personality | Conscientiousness High |  | 23 | 1.32 | 12 | 52.17 |  | 11 | 47.83 |  | 10 | 43.48 |  | 13 | 56.52 |  |
|  | Conscientiousness High | Conscientiousness High (code) | 16 | 0.92 | 9 | 56.25 | 0.04 | 7 | 43.75 | 0.03 | 8 | 50.00 | 0.03 | 8 | 50.00 | 0.03 |
|  |  | Active | 6 | 0.34 | 3 | 50.00 | 0.01 | 3 | 50.00 | 0.01 | 2 | 33.33 | 0.01 | 4 | 66.67 | 0.02 |
|  |  | Focused | 3 | 0.17 | 0 | 0.00 | 0 | 3 | 100.00 | 0.01 | 1 | 33.33 | 0 | 2 | 66.67 | 0.01 |
|  |  | Organized | 2 | 0.11 | 1 | 50.00 | 0 | 1 | 50.00 | 0 | 1 | 50.00 | 0 | 1 | 50.00 | 0 |
|  |  | Reflective | 3 | 0.17 | 3 | 100.00 | 0.01 | 0 | 0.00 | 0 | 1 | 33.33 | 0 | 2 | 66.67 | 0.01 |
|  |  | Responsible | 4 | 0.23 | 2 | 50.00 | 0.01 | 2 | 50.00 | 0.01 | 2 | 50.00 | 0.01 | 2 | 50.00 | 0.01 |
|  |  | Self-confident | 2 | 0.11 | 1 | 50.00 | 0 | 1 | 50.00 | 0 | 2 | 100.00 | 0.01 | 0 | 0.00 | 0 |
|  |  | Striving | 3 | 0.17 | 2 | 66.67 | 0.01 | 1 | 33.33 | 0 | 1 | 33.33 | 0 | 2 | 66.67 | 0.01 |
|  | Conscientiousness Low |  | 2 | 0.11 | 1 | 50.00 |  | 1 | 50.00 |  | 0 | 0.00 |  | 2 | 100.00 |  |
|  |  | Conscientiousness Low (code) | 2 | 0.11 | 1 | 50.00 | 0 | 1 | 50.00 | 0 | 0 | 0.00 | 0 | 2 | 100.00 | 0.01 |
|  |  | Distracted | 1 | 0.06 | 0 | 0.00 | 0 | 1 | 100.00 | 0 | 0 | 0.00 | 0 | 1 | 100.00 | 0 |
|  |  | Passive | 1 | 0.06 | 1 | 100.00 | 0 | 0 | 0.00 | 0 | 0 | 0.00 | 0 | 1 | 100.00 | 0 |
|  | Extraversion High |  | 8 | 0.46 | 5 | 62.50 |  | 3 | 37.50 |  | 6 | 75.00 |  | 2 | 25.00 |  |
|  |  | Extraversion High (code) | 8 | 0.46 | 5 | 62.50 | 0.02 | 3 | 37.50 | 0.01 | 6 | 75.00 | 0.03 | 2 | 25.00 | 0.01 |
|  |  | Extraverted | 4 | 0.23 | 2 | 50.00 | 0.01 | 2 | 50.00 | 0.01 | 3 | 75.00 | 0.01 | 1 | 25.00 | 0 |
|  |  | Sociable | 4 | 0.23 | 3 | 75.00 | 0.01 | 1 | 25.00 | 0 | 3 | 75.00 | 0.01 | 1 | 25.00 | 0 |
|  | Extraversion Low |  | 68 | 3.90 | 28 | 41.18 |  | 40 | 58.82 |  | 33 | 48.53 |  | 35 | 51.47 |  |
|  |  | Extraversion Low (code) | 67 | 3.84 | 27 | 40.30 | 0.1 | 40 | 59.70 | 0.16 | 32 | 47.76 | 0.12 | 35 | 52.24 | 0.14 |
|  |  | Independent | 5 | 0.29 | 4 | 80.00 | 0.02 | 1 | 20.00 | 0 | 4 | 80.00 | 0.02 | 1 | 20.00 | 0 |
|  |  | Introspective | 2 | 0.11 | 1 | 50.00 | 0 | 1 | 50.00 | 0 | 2 | 100.00 | 0.01 | 0 | 0.00 | 0 |
|  |  | Not very sociable | 5 | 0.29 | 3 | 60.00 | 0.01 | 2 | 40.00 | 0.01 | 3 | 60.00 | 0.01 | 2 | 40.00 | 0.01 |
|  |  | Shy/Introverted | 56 | 3.21 | 20 | 35.71 | 0.08 | 36 | 64.29 | 0.15 | 24 | 42.86 | 0.09 | 32 | 57.14 | 0.13 |
|  | Kindness High |  | 59 | 3.38 | 30 | 50.85 |  | 29 | 49.15 |  | 35 | 59.32 |  | 24 | 40.68 |  |
|  | Kindness High | Kindness High (code) | 46 | 2.64 | 22 | 47.83 | 0.09 | 24 | 52.17 | 0.1 | 26 | 56.52 | 0.11 | 20 | 43.48 | 0.08 |
|  |  | Altruism | 2 | 0.11 | 1 | 50.00 | 0 | 1 | 50.00 | 0 | 1 | 50.00 | 0 | 1 | 50.00 | 0 |
|  |  | Empathetic | 4 | 0.23 | 2 | 50.00 | 0.01 | 2 | 50.00 | 0.01 | 2 | 50.00 | 0.01 | 2 | 50.00 | 0.01 |
|  |  | Friendly | 9 | 0.52 | 5 | 55.56 | 0.02 | 4 | 44.44 | 0.02 | 7 | 77.78 | 0.03 | 2 | 22.22 | 0.01 |
|  |  | Helpful | 7 | 0.40 | 4 | 57.14 | 0.02 | 3 | 42.86 | 0.01 | 4 | 57.14 | 0.02 | 3 | 42.86 | 0.01 |
|  |  | Kind | 27 | 1.55 | 12 | 44.44 | 0.05 | 15 | 55.56 | 0.06 | 16 | 59.26 | 0.07 | 11 | 40.74 | 0.05 |
|  |  | Protector | 1 | 0.06 | 0 | 0.00 | 0 | 1 | 100.00 | 0 | 0 | 0.00 | 0 | 1 | 100.00 | 0 |
|  |  | Respectful | 3 | 0.17 | 2 | 66.67 | 0.01 | 1 | 33.33 | 0 | 2 | 66.67 | 0.01 | 1 | 33.33 | 0 |
|  |  | Sensitive | 6 | 0.34 | 4 | 66.67 | 0.02 | 2 | 33.33 | 0.01 | 3 | 50.00 | 0.01 | 3 | 50.00 | 0.01 |
|  | Kindness Low |  | 2 | 0.11 | 2 | 100.00 |  | 0 | 0.00 |  | 0 | 0.00 |  | 2 | 100.00 |  |
|  | Kindness Low | Kindness Low (code) | 1 | 0.06 | 1 | 100.00 | 0 | 0 | 0.00 | 0 | 0 | 0.00 | 0 | 1 | 100.00 | 0 |
|  |  | Antisocial | 1 | 0.06 | 1 | 100.00 | 0 | 0 | 0.00 | 0 | 0 | 0.00 | 0 | 1 | 100.00 | 0 |
|  |  | Apathetic | 1 | 0.06 | 1 | 100.00 | 0 | 0 | 0.00 | 0 | 0 | 0.00 | 0 | 1 | 100.00 | 0 |
|  | Neuroticism High |  | 32 | 1.83 | 14 | 43.75 |  | 18 | 56.25 |  | 15 | 46.88 |  | 17 | 53.13 |  |
|  | Neuroticism High | Neuroticism High (code) | 29 | 1.66 | 12 | 41.38 | 0.05 | 17 | 58.62 | 0.07 | 15 | 51.72 | 0.06 | 14 | 48.28 | 0.06 |
|  |  | Anxious | 1 | 0.06 | 0 | 0.00 | 0 | 1 | 100.00 | 0 | 0 | 0.00 | 0 | 1 | 100.00 | 0 |
|  |  | Compulsive | 1 | 0.06 | 0 | 0.00 | 0 | 1 | 100.00 | 0 | 0 | 0.00 | 0 | 1 | 100.00 | 0 |
|  |  | Dependent | 5 | 0.29 | 0 | 0.00 | 0 | 5 | 100.00 | 0.02 | 5 | 100.00 | 0.02 | 0 | 0.00 | 0 |
|  |  | Emotionally weak | 1 | 0.06 | 1 | 100.00 | 0 | 0 | 0.00 | 0 | 0 | 0.00 | 0 | 1 | 100.00 | 0 |
|  |  | Impatient | 1 | 0.06 | 1 | 100.00 | 0 | 0 | 0.00 | 0 | 0 | 0.00 | 0 | 1 | 100.00 | 0 |
|  |  | Inferiority complex | 2 | 0.11 | 0 | 0.00 | 0 | 2 | 100.00 | 0.01 | 1 | 50.00 | 0 | 1 | 50.00 | 0 |
|  |  | Insecure | 11 | 0.63 | 6 | 54.55 | 0.03 | 5 | 45.45 | 0.02 | 4 | 36.36 | 0.02 | 7 | 63.64 | 0.03 |
|  |  | Maladjusted | 6 | 0.34 | 4 | 66.67 | 0.02 | 2 | 33.33 | 0.01 | 3 | 50.00 | 0.01 | 3 | 50.00 | 0.01 |
|  |  | Mulish | 1 | 0.06 | 1 | 100.00 | 0 | 0 | 0.00 | 0 | 0 | 0.00 | 0 | 1 | 100.00 | 0 |
|  |  | Restless | 2 | 0.11 | 1 | 50.00 | 0 | 1 | 50.00 | 0 | 1 | 50.00 | 0 | 1 | 50.00 | 0 |
|  |  | Temperamental | 1 | 0.06 | 0 | 0.00 | 0 | 1 | 100.00 | 0 | 1 | 100.00 | 0 | 0 | 0.00 | 0 |
|  | Neuroticism Low |  | 3 | 0.17 | 2 | 66.67 |  | 1 | 33.33 |  | 1 | 33.33 |  | 2 | 66.67 |  |
|  | Neuroticism Low | Personality Neuroticism Low (code) | 3 | 0.17 | 2 | 66.67 | 0.01 | 1 | 33.33 | 0 | 1 | 33.33 | 0 | 2 | 66.67 | 0.01 |
|  |  | Calm | 2 | 0.11 | 1 | 50.00 | 0 | 1 | 50.00 | 0 | 0 | 0.00 | 0 | 2 | 100.00 | 0.01 |
|  |  | Patient | 1 | 0.06 | 1 | 100.00 | 0 | 0 | 0.00 | 0 | 1 | 100.00 | 0 | 0 | 0.00 | 0 |
|  | Openness to experience High |  | 53 | 3.04 | 25 | 47.17 |  | 28 | 52.83 |  | 28 | 52.83 |  | 25 | 47.17 |  |
|  | Openness to experience High | Personality Openness to experience High (code) | 49 | 2.81 | 22 | 44.90 | 0.09 | 27 | 55.10 | 0.11 | 26 | 53.06 | 0.1 | 23 | 46.94 | 0.09 |
|  |  | Ambitious | 6 | 0.34 | 4 | 66.67 | 0.02 | 2 | 33.33 | 0.01 | 3 | 50.00 | 0.01 | 3 | 50.00 | 0.01 |
|  |  | Curious | 38 | 2.18 | 15 | 39.47 | 0.06 | 23 | 60.53 | 0.1 | 20 | 52.63 | 0.08 | 18 | 47.37 | 0.07 |
|  |  | Funny | 3 | 0.17 | 1 | 33.33 | 0 | 2 | 66.67 | 0.01 | 2 | 66.67 | 0.01 | 1 | 33.33 | 0 |
|  |  | Imaginative/Intuitive | 3 | 0.17 | 3 | 100.00 | 0.01 | 0 | 0.00 | 0 | 2 | 66.67 | 0.01 | 1 | 33.33 | 0 |
|  |  | Logic | 1 | 0.06 | 1 | 100.00 | 0 | 0 | 0.00 | 0 | 1 | 100.00 | 0 | 0 | 0.00 | 0 |
|  |  | Perceptive | 1 | 0.06 | 0 | 0.00 | 0 | 1 | 100.00 | 0 | 0 | 0.00 | 0 | 1 | 100.00 | 0 |
|  |  | Tolerant | 1 | 0.06 | 1 | 100.00 | 0 | 0 | 0.00 | 0 | 0 | 0.00 | 0 | 1 | 100.00 | 0 |
|  | Openness to experience Low |  | 1 | 0.06 | 0 | 0.00 |  | 1 | 100.00 |  | 1 | 100.00 |  | 0 | 0.00 |  |
|  | Openness to experience Low | Personality Openness to experience Low (code) | 1 | 0.06 | 0 | 0.00 | 0 | 1 | 100.00 | 0 | 1 | 100.00 | 0 | 0 | 0.00 | 0 |
|  |  | Conservatism | 1 | 0.06 | 0 | 0.00 | 0 | 1 | 100.00 | 0 | 1 | 100.00 | 0 | 0 | 0.00 | 0 |
| Self-perceptions | Self-perceptions |  | 6 | 0.34 | 1 | 16.67 |  | 5 | 83.33 |  | 2 | 33.33 |  | 4 | 66.67 |  |
|  |  | Low self-esteem | 5 | 0.29 | 0 | 0.00 | 0 | 5 | 100.00 | 0.02 | 1 | 20.00 | 0 | 4 | 80.00 | 0.02 |
|  |  | Not aware of her/his own attractiveness | 1 | 0.06 | 1 | 100.00 | 0 | 0 | 0.00 | 0 | 1 | 100.00 | 0 | 0 | 0.00 | 0 |
| Skills/Ability | Academic |  | 18 | 1.03 | 9 | 50.00 |  | 9 | 50.00 |  | 6 | 33.33 |  | 12 | 66.67 |  |
|  |  | High academic performance | 11 | 0.63 | 5 | 45.45 | 0.02 | 6 | 54.55 | 0.03 | 3 | 27.27 | 0.01 | 8 | 72.73 | 0.03 |
|  |  | Low academic performance | 4 | 0.23 | 2 | 50.00 | 0.01 | 2 | 50.00 | 0.01 | 2 | 50.00 | 0.01 | 2 | 50.00 | 0.01 |
|  |  | Need help to exploit his/her abilities | 1 | 0.06 | 1 | 100.00 | 0 | 0 | 0.00 | 0 | 1 | 100.00 | 0 | 0 | 0.00 | 0 |
|  |  | No skilled in art activities | 1 | 0.06 | 1 | 100.00 | 0 | 0 | 0.00 | 0 | 0 | 0.00 | 0 | 1 | 100.00 | 0 |
|  |  | Standard ability | 1 | 0.06 | 0 | 0.00 | 0 | 1 | 100.00 | 0 | 0 | 0.00 | 0 | 1 | 100.00 | 0 |
|  | Coping |  | 4 | 0.23 | 2 | 50.00 |  | 2 | 50.00 |  | 1 | 25.00 |  | 3 | 75.00 |  |
|  |  | Ignoring classmates' mockery | 1 | 0.06 | 1 | 100.00 | 0 | 0 | 0.00 | 0 | 0 | 0.00 | 0 | 1 | 100.00 | 0 |
|  |  | Masking | 3 | 0.17 | 1 | 33.33 | 0 | 2 | 66.67 | 0.01 | 1 | 33.33 | 0 | 2 | 66.67 | 0.01 |
|  | Intellectual/cognitive |  | 58 | 3.33 | 37 | 63.79 |  | 21 | 36.21 |  | 34 | 58.62 |  | 24 | 41.38 |  |
|  |  | Able in different knowledge areas | 5 | 0.29 | 5 | 100.00 | 0.02 | 0 | 0.00 | 0 | 2 | 40.00 | 0.01 | 3 | 60.00 | 0.01 |
|  |  | Concentration | 3 | 0.17 | 2 | 66.67 | 0.01 | 1 | 33.33 | 0 | 1 | 33.33 | 0 | 2 | 66.67 | 0.01 |
|  |  | Creative | 2 | 0.11 | 2 | 100.00 | 0.01 | 0 | 0.00 | 0 | 2 | 100.00 | 0.01 | 0 | 0.00 | 0 |
|  |  | Disability | 1 | 0.06 | 0 | 0.00 | 0 | 1 | 100.00 | 0 | 0 | 0.00 | 0 | 1 | 100.00 | 0 |
|  |  | Fast in combining clothes | 1 | 0.06 | 1 | 100.00 | 0 | 0 | 0.00 | 0 | 1 | 100.00 | 0 | 0 | 0.00 | 0 |
|  |  | Intelligent | 44 | 2.52 | 27 | 61.36 | 0.11 | 17 | 38.64 | 0.07 | 26 | 59.09 | 0.11 | 18 | 40.91 | 0.07 |
|  |  | More skilled in theoretical subjects than practical or sports | 1 | 0.06 | 0 | 0.00 | 0 | 1 | 100.00 | 0 | 1 | 100.00 | 0 | 0 | 0.00 | 0 |
|  |  | No Disability | 1 | 0.06 | 0 | 0.00 | 0 | 1 | 100.00 | 0 | 1 | 100.00 | 0 | 0 | 0.00 | 0 |
|  | Socio-emotional |  | 35 | 2.01 | 15 | 42.86 |  | 20 | 57.14 |  | 19 | 54.29 |  | 16 | 45.71 |  |
|  |  | Good at talking | 1 | 0.06 | 0 | 0.00 | 0 | 1 | 100.00 | 0 | 1 | 100.00 | 0 | 0 | 0.00 | 0 |
|  |  | Great emotional intelligence | 1 | 0.06 | 1 | 100.00 | 0 | 0 | 0.00 | 0 | 0 | 0.00 | 0 | 1 | 100.00 | 0 |
|  |  | Low social skills | 31 | 1.78 | 14 | 45.16 | 0.06 | 17 | 54.84 | 0.07 | 17 | 54.84 | 0.07 | 14 | 45.16 | 0.06 |
|  |  | Standard social skills | 2 | 0.11 | 0 | 0.00 | 0 | 2 | 100.00 | 0.01 | 1 | 50.00 | 0 | 1 | 50.00 | 0 |
| Social | Social |  | 35 | 2.01 | 13 | 37.14 |  | 22 | 62.86 |  | 15 | 42.86 |  | 20 | 57.14 |  |
|  |  | Bullying | 2 | 0.11 | 1 | 50.00 | 0 | 1 | 50.00 | 0 | 0 | 0.00 | 0 | 2 | 100.00 | 0.01 |
|  |  | Few friends | 6 | 0.34 | 2 | 33.33 | 0.01 | 4 | 66.67 | 0.02 | 3 | 50.00 | 0.01 | 3 | 50.00 | 0.01 |
|  |  | Freak/Nerd | 7 | 0.40 | 3 | 42.86 | 0.01 | 4 | 57.14 | 0.02 | 0 | 0.00 | 0 | 7 | 100.00 | 0.03 |
|  |  | Many friends | 1 | 0.06 | 0 | 0.00 | 0 | 1 | 100.00 | 0 | 1 | 100.00 | 0 | 0 | 0.00 | 0 |
|  |  | Misunderstood | 2 | 0.11 | 1 | 50.00 | 0 | 1 | 50.00 | 0 | 1 | 50.00 | 0 | 1 | 50.00 | 0 |
|  |  | Not popular | 7 | 0.40 | 2 | 28.57 | 0.01 | 5 | 71.43 | 0.02 | 3 | 42.86 | 0.01 | 4 | 57.14 | 0.02 |
|  |  | Rejected | 10 | 0.57 | 4 | 40.00 | 0.02 | 6 | 60.00 | 0.03 | 7 | 70.00 | 0.03 | 3 | 30.00 | 0.01 |
| *Note*: *k*: frequency of codifications. *c*: co-occurrence coefficient. | | | | | | | | | | | | | | | | |
